# Genetic variations analysis for complex brain disease diagnosis using machine learning techniques: opportunities and hurdles

**DOI:** 10.7717/peerj-cs.697

**Published:** 2021-09-20

**Authors:** Hala Ahmed, Louai Alarabi, Shaker El-Sappagh, Hassan Soliman, Mohammed Elmogy

**Affiliations:** 1 Information Technology Department, Faculty of Computers and Information, Mansoura University, Mansoura, Egypt; 2 Department of Computer Science, Umm Al-Qura University, Makkah, Saudi Arabia; 3 Centro Singular de Investigación en Tecnoloxías Intelixentes (CiTIUS), Universidade de Santiago de Compostela, Santiago de Compostela, Spain; 4 Information Systems Department, Faculty of Computers and Artificial Intelligence, Benha University, Benha, Egypt

**Keywords:** Genetic analysis, Brain disease, Machine learning, Deep learning, Single nucleotide polymorphism (SNP), Microarrays

## Abstract

**Background and Objectives:**

This paper presents an in-depth review of the state-of-the-art genetic variations analysis to discover complex genes associated with the brain’s genetic disorders. We first introduce the genetic analysis of complex brain diseases, genetic variation, and DNA microarrays. Then, the review focuses on available machine learning methods used for complex brain disease classification. Therein, we discuss the various datasets, preprocessing, feature selection and extraction, and classification strategies. In particular, we concentrate on studying single nucleotide polymorphisms (SNP) that support the highest resolution for genomic fingerprinting for tracking disease genes. Subsequently, the study provides an overview of the applications for some specific diseases, including autism spectrum disorder, brain cancer, and Alzheimer’s disease (AD). The study argues that despite the significant recent developments in the analysis and treatment of genetic disorders, there are considerable challenges to elucidate causative mutations, especially from the viewpoint of implementing genetic analysis in clinical practice. The review finally provides a critical discussion on the applicability of genetic variations analysis for complex brain disease identification highlighting the future challenges.

**Methods:**

We used a methodology for literature surveys to obtain data from academic databases. Criteria were defined for inclusion and exclusion. The selection of articles was followed by three stages. In addition, the principal methods for machine learning to classify the disease were presented in each stage in more detail.

**Results:**

It was revealed that machine learning based on SNP was widely utilized to solve problems of genetic variation for complex diseases related to genes.

**Conclusions:**

Despite significant developments in genetic diseases in the past two decades of the diagnosis and treatment, there is still a large percentage in which the causative mutation cannot be determined, and a final genetic diagnosis remains elusive. So, we need to detect the variations of the genes related to brain disorders in the early disease stages.

## Introduction

The genetic mechanisms of complex diseases are challenging to discover. The power to identify the set of genes responsible for complex diseases using current methods is often lacking. In the last few years, large quantities of biological data have been generated for genomics and proteomics with rapid developments (Mezlini & Goldenberg, 2017). These data need a complex mathematical analysis to interpret biological data using the interdisciplinary science composed of computer science and information technology, which is known as bioinformatics or computational biology. This new research area is more important and will grow rapidly as we continue producing and integrating large amounts of protein, genomic and other data.

Bioinformatics research is an active area to solve biological problems. It utilizes data mining (DM) techniques and its applications to analyze biological data. In medicine, DM is emerging of high importance for diagnosing neurodegenerative diseases and supporting prognosis to provide deeper understanding (Joshi et al., 2010). The data analysis includes many examples, such as protein structure prediction, gene classification, gene classification based on microarray data, and clustering gene expression data. Therefore, DM and bioinformatics interaction should be increased to provide great potential (Association, 2019).

The association of the structural changes with brain diseases, which occurs as the disease progresses, is unknown (Sherif, Zayed & Fakhr, 2015; Kim et al., 2013; Bertram, Lill & Tanzi, 2010; Yokoyama et al., 2015; Hwang et al., 2014; Guerreiro & Hardy, 2012). So, we need to identify many genetic markers about their association with brain diseases. In the 1950s, artificial intelligence (AI) appeared as an independent field because of the potentials to make machines intelligent like a human. In streamlining complex analytical workflows, bioinformatics with AI techniques play an essential role in performing multistep analysis within one analytical framework. The problems with biological data, such as the complexity of data and the growing exponential rate, can be solved with workflows that enable processing and analysis. Machine learning (ML), knowledge discovery, and reasoning deployed by AI techniques are continuously improving. A formidable combination is presented between bioinformatics and AI, where the analysis of complex biological systems is enabled by bioinformatics and human-like reasoning. We can perform complex tasks based on reasoning by using AI-based tools (Sandraa et al., 2010; Bertram & Tanzi, 2012).

In the past few years, intensive computational efforts have been performed to study single nucleotide polymorphisms (SNPs) structural and functional consequences. In this context, ML and DM techniques have been widely performed for SNP data analysis. Biological systems are complex, so most studies on large scales concentrate only on one specific aspect of the biological system. For example, genome-wide association studies (GWAS) concentrates on genetic variants associated with phenotypes measurement. So, the feature should be chosen precisely from the provided dataset. The best possible subset of the feature sets is determined using search methods. Then, evaluation techniques are used to evaluate them.

Some examples of genetic diseases that affect the brain are Alzheimer’s Disease (AD) and Parkinson’s, as shown in Table 1. The progressive decline of cognition and memory is caused by AD, which is a degenerative disease. It causes the nerve cells’ degeneration in the brain due to AD’s side effects, which are related to language and memory (Ang et al., 2015). After 65 years, symptoms appear, and the spread of disease with age increases sharply. It is considered the most common form of dementia in the disease’s onset genetic factors for specific genes. However, they are not the primary effect of the disease. Also, the suffering can be increased by other factors, such as age, smoking and alcohol (Ang et al., 2015; Zuk et al., 2012, 2014; Hormozdiari et al., 2015). AD has many common symptoms, such as complete memory loss, impairments of movements, misplacing things, verbal communication, and abnormal moods (Sherif, Zayed & Fakhr, 2015). If AD is not initially diagnosed, the disease’s severity increases (Hemani, Knott & Haley, 2013; Pinto et al., 2014; Parikshak, Gandal & Geschwind, 2015; Nakka, Raphael & Ramachandran, 2016).

**Table 1:** Early symptoms comparison of various brain diseases.

Comparison between early symptom	Dementia of Lewy body	Parkinson’s disease (PD)	Alzheimer’s disease (AD)
Age of onset	>60 years old	>70 years old	>60 years old
Gender-specific	Men > Women	Conflicting	Men = women
Family history	No	Conflicting	Yes
Significant Loss of Memory	Possible	Possible Years After Diagnosis	Always
Problems of Language	Possible	Possible	Possible
Fluctuating Cognitive Abilities	Likely	Possible	Possible
Planning or Problem-solving Abilities	Likely	Possible	Possible
Decline in Thinking Abilities that Interfere with Everyday Life	Always	Possible Years After Diagnosis	Always
Difficulty with a Sense of Direction or Spatial Relationships between Objects	Likely	Possible	Possible

One of the most demanding tasks in a post-genomic era is identifying disease genes from a vast amount of genetic data. Moreover, complex diseases present a very heterogeneous genotype that makes it difficult to identify biological markers. ML is widely used to identify these markers, but their performance relies heavily on the size and quality of the data available (Asif et al., 2018). Also, modern computational systems help researchers to analyze complex data like genetic information of humans and its underlying patterns. These patterns reveal the genes that cause diseases. Mathematical models are helpful to build robust ML models for analyzing gene expression. So, this paper introduces a comprehensive review of genetic variations analysis for discovering complex disease-related genes, especially genetic brain diseases. The SNPs data is commonly used as a marker-based association or association study in a population. We focus on identifying a disease’s biomarkers based on SNPs that alter phenotypes by altering some molecular functions. There are many challenges to defining so-called functional variants. First, the marker variants themselves may be unbalanced (or linkage, depending on the study) with the causal variant. Second, the challenge is identifying candidate genes related to disease to narrow down an area for SNP prioritization. Finally, the molecular function is poorly understood, so we need to increase how SNPs disrupt their functionality (Hemani, Knott & Haley, 2013; Pinto et al., 2014; Parikshak, Gandal & Geschwind, 2015; Nakka, Raphael & Ramachandran, 2016; Guyon et al., 2002; Printy et al., 2014).

**Figure 1:**
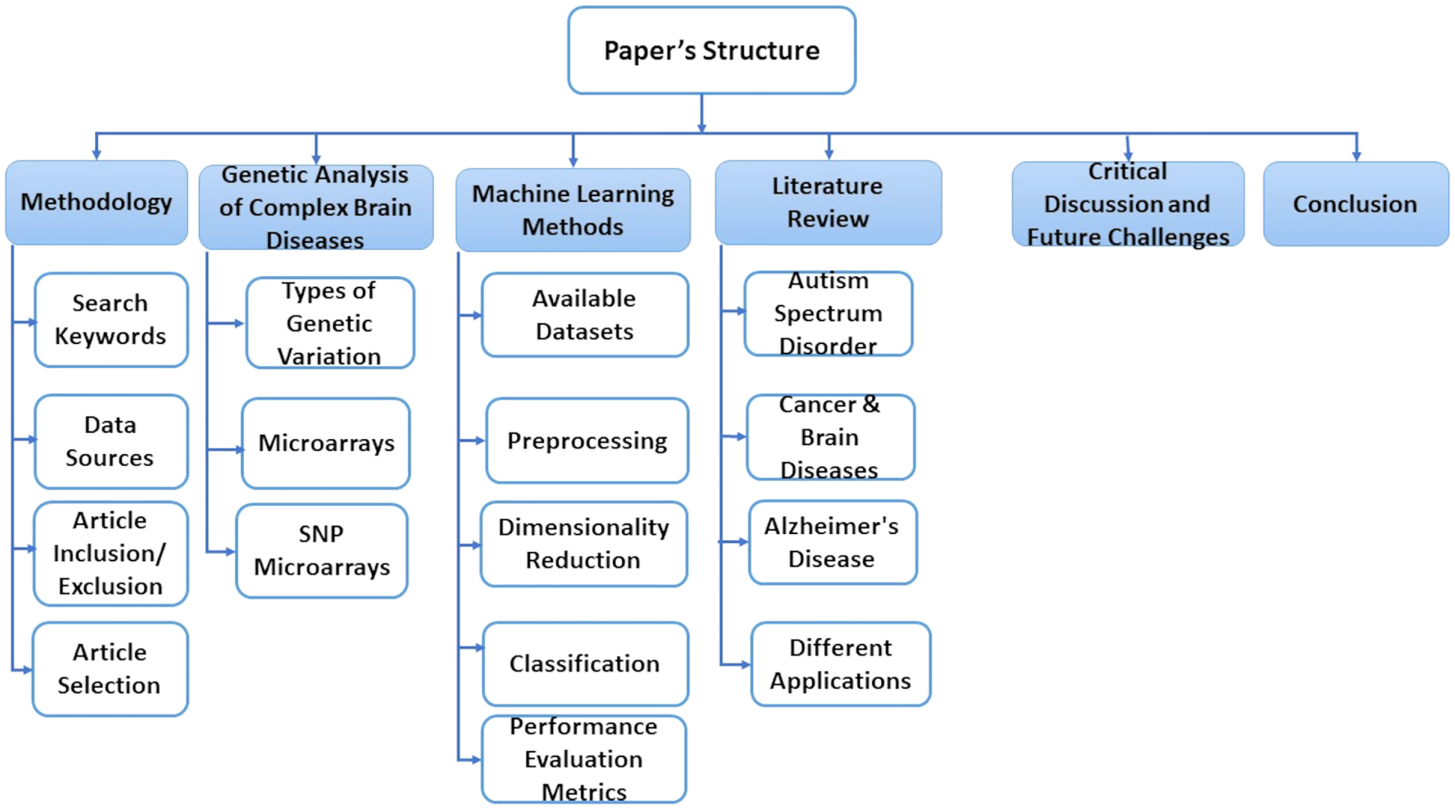
The structure of the survey.

This paper is consisting of six sections. In “Methodology”, we explain some basic concepts of gene sequence and types of genetic variations. In “A Genetic Analysis of Complex Brain Diseases”, we present some classification, preprocessing, and dimensionality reduction techniques. “ML Methods” discusses essential research areas about various diseases depending on discovering genetic mechanisms as shown in tables in this section. “Literature Review on Complex Disease & Applications” introduces current research topics, future directions, and challenges. Finally, we discuss the conclusion of this work in “Critical Discussion and Future Challenges”. Also, Fig. 1 represents the structure of these sections.

## Methodology

**Figure 2:**
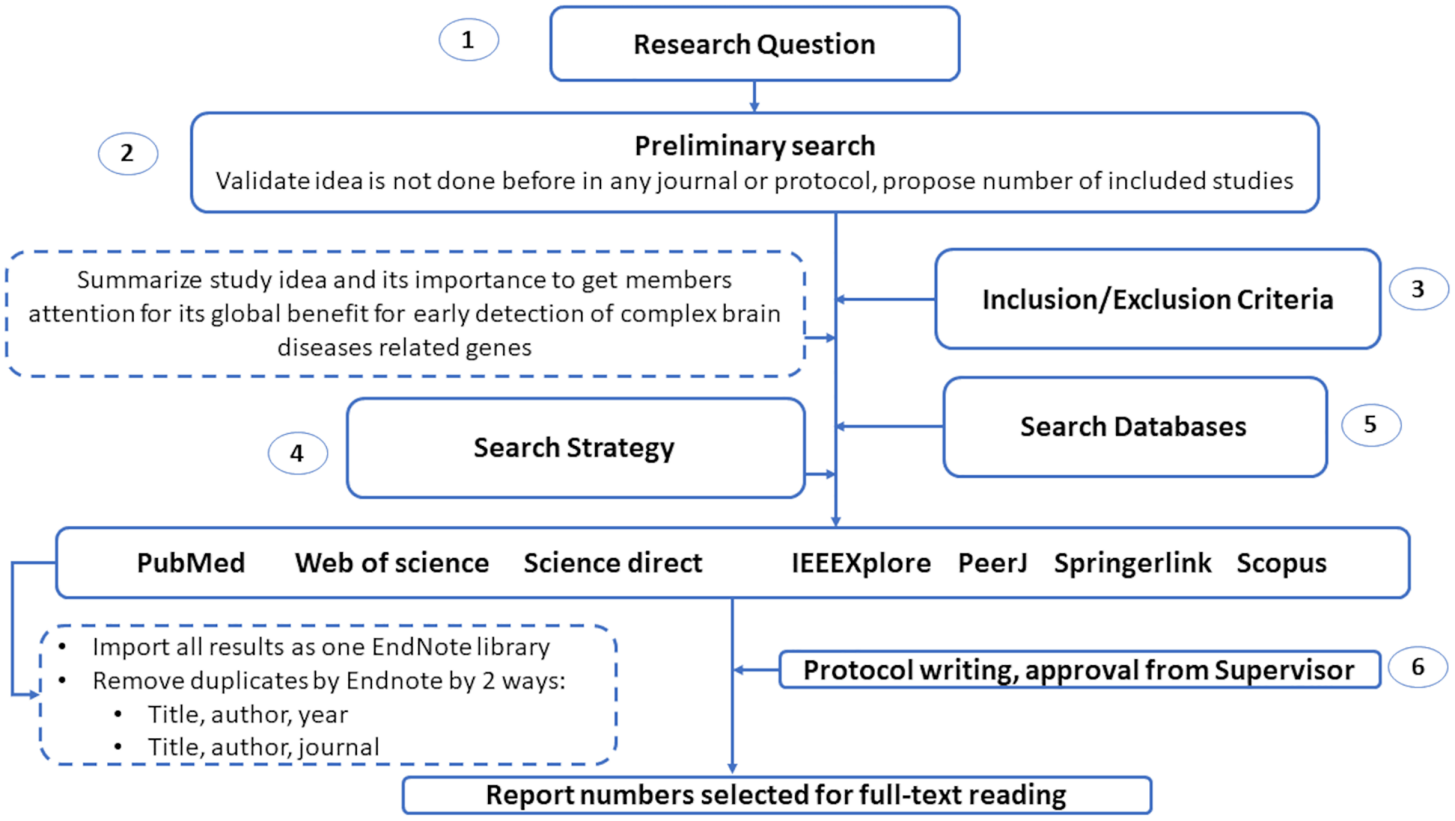
The guidelines for systematic review analysis.

This section introduces the protocol used to survey various genetic variations to diagnose complex brain diseases using machine learning. We list the search keywords, data sources, inclusion/exclusion criteria, and article selection in this section. Also, we illustrated in Fig. 2 the structure of the systematic review analysis and how to remove the overlap articles among different databases. We remove overlap with two ways by Endnote by choosing the following elements: (1) Title, author and year. (2) Title, author and journal.

### Search keywords

Keywords have been carefully chosen for the initial search. After the initial search, new words found in several relevant articles have formulated multiple keywords. The study’s keywords included “genetic analysis for brain disease using machine learning”, “genetic variations based on single nucleotide polymorphism (SNP)”, “Gene analysis for complex brain disease based on machine learning”.

### Data sources

Our survey used different academic database sources to obtain articles, as shown in Table 2.

**Table 2:** The used databases for selecting the academic articles in this article.

Academic database	Source
Science direct	http://www.sciencedirect.com/
Springerlink	https://link.springer.com/
IEEEXplore	https://ieeexplore.ieee.org/
Web of science	https://apps.webofknowledge.com/
PubMed	https://pubmed.ncbi.nlm.nih.gov/
PeerJ	https://peerj.com/
Scopus	https://www.scopus.com/

### Article inclusion/exclusion criteria

Inclusion/exclusion criteria have been developed to determine which papers are eligible for the next review phase. Relevance for research has been considered for articles that meet inclusion criteria and excluded articles that do not fulfill inclusion criteria. The following criteria are defined for inclusion/exclusion.

### Inclusion

1. Our paper only focuses on genetic variation for complex diseases related to genes.
2. Only articles that performed on genetic variation for complex diseases related to genes using machine learning.
3. For inclusion, only articles in English were taken into account.

### Exclusion

1. Papers that do not focus on genetic variation for complex diseases related to genes were excluded.
2. Articles that do not concentrate on genetic variation for complex diseases related to genes using machine learning were excluded.
3. Articles that are not in English were excluded.

### Article selection

Research has established criteria for inclusion and exclusion to choose which articles are eligible for the next review phase. Articles that satisfy the inclusion criteria were considered research-related, and items that did not meet the inclusion criteria were excluded. In the previous section, we present the list of inclusion/exclusion criteria. Three phases were followed to select an article for this research. The first step was to consider extracting only the titles and abstracts of the articles. The second stage was to analyze the abstract, introduction, and conclusion to refine the first stage’s choice. At the end of the process, the articles were carefully read and the quality of the papers was then measured according to their relevance to the research.

## A Genetic Analysis of Complex Brain Diseases

The core of modern medical genetics is to enhance our understanding of the genetic mutations and possible factors in genetic risk, which cause or contribute directly to human disease. Evolutionary theory can describe selective forces that affect causal alleles and susceptibility to human genetic disorders. In the general population, complex disorders are common and result from the interaction of many sites of sensitivity and environmental factors (Rahit & Tarailo-Graovac, 2020; Spataro et al., 2017). In contrast, Mendelian disorders are usually rare and have predictable genotypes as they are generally caused by a single causative mutation in the gene. However, the classic distinction between Mendelian diseases and complex diseases is not always absolute due to its heterogeneity and incomplete penetration. There is a continuum between pure Mendelian diseases and more complex diseases.

Diseases that follow Mendelian inheritance patterns are called Mendelian disorders. About 80% of all rare diseases are hereditary, and most of these diseases are monogenic/Mendelian. According to an estimate, there are 400 million people worldwide with around 7,000 various rare diseases (Rahit & Tarailo-Graovac, 2020; Spataro et al., 2017). So, studying gene function by biomedical researchers should consider the impact of the variations between these diseases. Genetic information about these diseases can supply valuable insight into the critical regions and operating ranges, proteins, or regulatory elements. Genetic variants may differ in nature concerning single nucleotide variants, tandem repeats or deletions, and small insertions to massive copy number variants (CNVs) (Siavelis et al., 2016). Until lately, genetic variation information is limited to some extent. However, many large-scale surveys of diversity have provided numerous data on covariance. A picture is started from the driving forces in human evolution and population diversity that is emerged (Siavelis et al., 2016; Mathur, 2018; Raza, 2012; Wodehouse, 2006; Mount & Pandey, 2005; Barnes, 2010).

**Figure 3:**
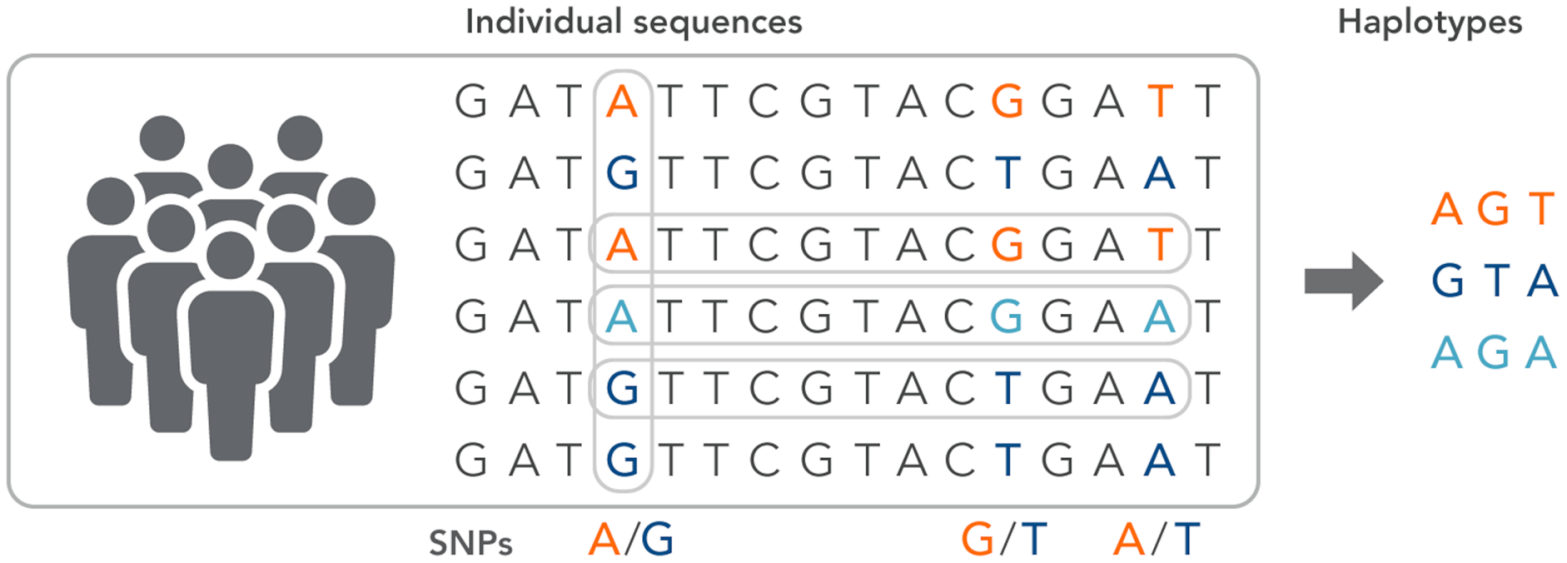
The haplotype of SNPs for individuals.

SNPs work as a pointer in the association and linkage studies to identify the genome’s part in a particular disease (Hasnain et al., 2020; Ng & Henikoff, 2003; Uppu, Krishna & Gopalan, 2016; Liang & Kelemen, 2008; Korani et al., 2019). Polymorphs are found in the same coding and organizing regions, which may be involved in diseases. A non-synonymous SNP is called the SNP that causes amino acid substitution. The amino acid variations that lead to genetic lesions that cause diseases have a strong concentration and interest. Studies aimed at determining polymorphisms and analysis of mutation complement each other to identify substitutions for amino acids in protein code regions where any variation can change protein function or structure (Clare & King, 2001). Substitution of one nucleotide by another is the most simple form of DNA variation between individuals. As shown in Fig. 3, this type of change is known as SNP. SNPs are estimated to occur at a rate of 1 in 1,000 bps across the genome (Shastry, 2007). These simple changes can be of transitional or transitional type. Approximately 50% of the polymorphisms are found in non-coding regions, whereas 25% of them result in missense mutations (encoding SNPs or cSNPs). The remaining 25% are silent mutations that do not alter the code of the amino acid. These silent SNPs are known as synonymous, and most likely, they are not subject to natural selection.

On the other side, non-synonymous SNPs may cause pathology and may be natural for selection (nSNPs and codified amino acids). SNPs (both synonymous and unknown) influence promoter activity and pre-mRNA morphology (or stability) (Shastry, 2007). They also change the protein’s ability to bind to its substrates or inhibitors and alter subcellular proteins’ localization (nSNPs). Therefore, they may be responsible for disease susceptibility, drug deposition, and genome evolution. However, many of them affect gene function. Besides, many of them are not harmful to living organisms and must have survived selection pressure (Halushka et al., 1999).

Due to the inheritance of certain diseases, a direct aim of the human genome project is to identify the genes which predispose individuals to different diseases. It also examines how sequential variation of a gene affects the functions of its product. SNPs often occur across the genome, as has already been mentioned. They can therefore be used as markers by studying correlation to discover disease-causing genes. Two closely related alleles (gene and marker) are supposed to be inherited together in such studies. So, a simple comparison of patterns of genetic differences between patients and normal individuals may facilitate a method for determining which sites are responsible for disease susceptibility.

### Types of genetic variation

Candidate genes tend to decrease the number of SNPs in the study to some mutations where genes are intended to provide the genetic basis for the disease under study. Although overall candidate genes’ data sets are considered to be, the various tests of hundreds or even thousands of polymorphisms in our situation make link detection troublesome. A conceivable method to overcome testing overpowering quantities of SNPs, mainly based on the studies of a candidate gene, is to order SNPs as per their priority of functional significance (Clare & King, 2001). Previous natural information can be used in current databases to reduce the number of SNPs by focusing on specific genomic regions, and computational methodologies and aptitude are used to separate the neutral polymorphisms from poly-morphisms of potential functional interest (Mikhail et al., 2020).

The critical biological determinant is a genetic variation that supports evolution and determines the phenotype’s genetic basis. A researcher may wish to deal with the genetic data depending on the researcher’s viewpoint (Barnes, 2010). Researchers of the biomedical view tend to focus on genetic or phenotypic directions. The function of a gene cannot be understood fully without realizing the possible variation within the gene. This means that the biomedical researchers, who study genetics, must know what variants present and what effect these variants are on the gene’s function and thus the phenotype. The genetic variation comes in many forms, but every form arises from only two types of mutation events. The simplest type of different genetic variation results from a simple primary substitution that comes from mutation. This type of mutation event explains the most common form of variation (Barnes, 2010), SNP. However, it also represents rare mutations that may manifest Mendelian inheritance in families. Most other kinds of variation arise directly or indirectly from inserting or deleting a portion of DNA.

### Microarrays

Microarray is a technique in which 1,000 nucleic acids are attached to a surface. They are used to measure DNA sequences’ concentration in a mixture by hybridization and the subsequent detection of hybridization events (Bumgarner, 2013). Millions of sequences in one reaction are simultaneously analyzed using microarrays. Microarrays come in three basic types: (A) spotted arrays on glass, (B) self-assembled arrays and (C) insitu synthesized arrays. Many types of microarrays depend on the type of data (Berry et al., 2019; Mao, Young & Lu, 2007; Prince, 2017; Smith et al., 2007; Isik, 2010; Yin et al., 2017). These types are DNA microarray, SNP microarray, cDNA microarrays, SNP microarrays, protein microarrays, MM chips, peptide microarrays, etc.

All studies on genome association aim to determine the genetic basis for traits and disease sensitivities using SNP microarrays. They carry the genetic variants, which are the most common in the human. Variants were identified for families at risk for several diseases (Coelho et al., 2009; Chen et al., 2012). However, with only a few notable exceptions, such as the related age of macular degeneration, risk variants usually describe only a small portion of the genetic risks known for their existence. Many factors are found that favor is contributing to this observation. Common variants may have minor effects on the phenotype or have variable penetration due to cognitive or epigenetic effects. Two other factors are CNVs and rare variants (Barnes, 2010). Genomic variation types have an essential effect. However, examine these variants on disease phenotypes should perform.

### SNP microarrays

SNP microarray is a type of DNA microarrays, which is used to identify polymorphisms within a population. SNP is a variation of a single site in DNA, and it is the most common kind of variation in the genome. About 335 million polymorphisms have been specified in the human genome (Berry et al., 2019; Mao, Young & Lu, 2007). Microarrays of SNP have grown as a robust tool for the large-scale detection of epigenetic changes in genomes of predictive and/or predictive value. For genotyping in 1998; SNP matrix technology was developed (Association, 2019). Since then, this technique has been extensively developed and has become one of the most robust genetic analyses (Latkowski & Osowski, 2015; González & Belanche, 2013; Xue, Zhang & Browne, 2012; Teng, Dong & Zhou, 2017; Kong, Mou & Yang, 2009; Saeys, Inza & Larranaga, 2007). In this study, the very long execution times result in an enormous volume of data. Some preliminary tests were performed to select the three following constraints for the primary sequence of experiments (Mao, Young & Lu, 2007). First, it depends on the used data, which we could not generalize all data analysis. The second issue is the selection of SNP. Finally, the third issue is the used ML method.

## ML Methods

**Figure 4:**
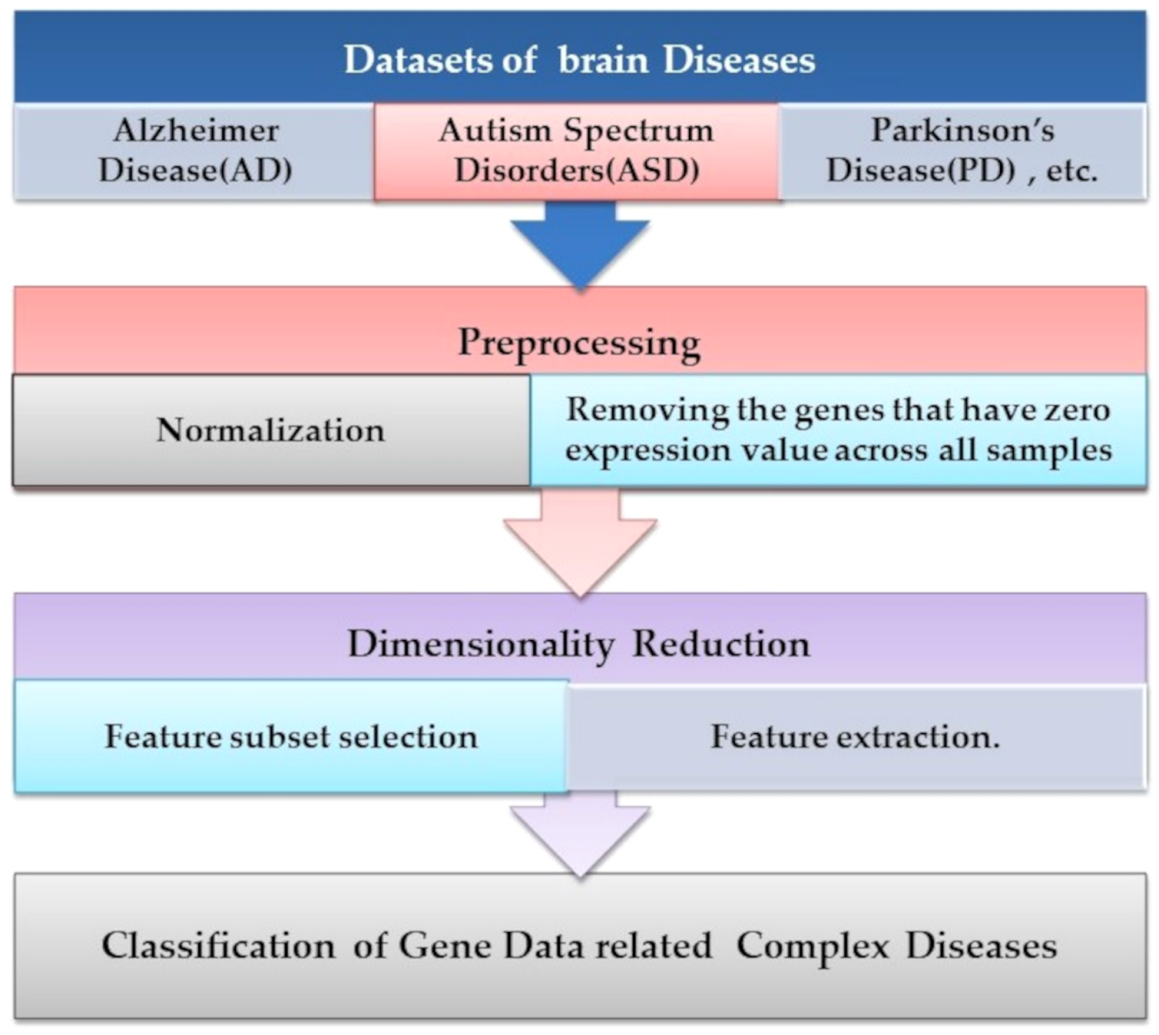
The common ML procedure for the complex brain disease diagnosis.

Microarray technology is a valuable tool for capturing information from biological, genetic data. An extensive set of genetic data and many computational techniques are needed to determine whether a human is normal or abnormal as we need to identify the biomarker in the gene responsible for the diseases. ML algorithms play an important role in distinguishing unhealthy and normal genes extracted from humans’ genomes (Karthik & Sudha, 2018). ML methods contain steps to manage the classification process. ML methods contain some stages to manage the diagnosis process and determine candidate genes that cause diseases, as shown in Fig. 4. Each step is discussed in the following subsections.

### Available datasets

In this section, we provided several benchmark databases that can be used to test the researchers’ proposed techniques and methods (Guyon et al., 2002) as shown in Table 3. Research in biology and medicine may benefit from higher-order gene screening to confirm recent genetic disease research discoveries or new ways to explore them. Therefore, the ADNI (Rémi et al., 2011) dataset’s primary goal is to support researchers with the chance to combine genetics, imaging, and clinical data with supporting the investigation of disease mechanisms. Genotyping and sequencing data were generated for ADNI 1, ADNI GO, and ADNI 2 subjects and are available to ADNI investigators. ADNI was released in 2003 as a public-private partnership. ADNI phase 1 data were collected from 757 subjects (214 controls, 366 MCI and 177 AD cases).

**Table 3:** Some of the benchmark datasets.

Dataset	Diseases	Source
GEO Database	ASD	https://www.ncbi.nlm.nih.gov/geo/
(KEGG) database	AD	https://www.genome.jp/kegg/
ADNI/Whole-genome sequencing (WGS) datasets	AD	http://adni.loni.usc.edu/
TCGA	Cancer	The Cancer Genome Atlas (TCGA): https://portal.gdc.cancer.gov/
UCI for Cancer	Cancer	UCI Machine Learning Repository: http://archive.ics.uci.edu/ml
NCBI Gene Expression Omnibus (GEO)	Cancer	NCBI repository: https://www.ncbi.nlm.nih.gov/geo/

### Preprocessing

There is a lot of information in a raw dataset prepared by researchers. It depends entirely on the requirements and purposes if the information is valuable or not. Data preprocessing is the first step in ML. It is necessary to ensure that the dataset is fully adapted to the needs. First, we removed unwanted attributes, missing values, and correct arrangement, and the expression value is normalized. The Bioconductor package completes the two steps. This raw file must be processed to extract the correct attributes for the following study. R programming language is chosen as a preferred language for analyzing data. Therefore, R programming language packages were applied for the preprocessing of the dataset. The Bioconductor analyzed the value of the expression and set the data set further, using the data normalization that reduces the data range to be studied as shown in Eq. (1). Unwanted attributes and the samples that have missing values must be removed. Data rearrangement according to format requirements will be completed before the next phases proceed. For classification purposes, only desired attributes are kept.

Gene removal with no expression value through all samples is one of the most simple and straightforward preprocessing techniques used in Karthik & Sudha (2018) and Daoud & Mayo (2019). Researchers applied this step by removing samples with 20% of the deleted features. After then, the data are normalized. Normalization is to remove the unimportant technical differences in data and to filter matrices with the *p*-value. Preprocessing is a step towards facilitating the use of data. The normalization was performed in the preprocessing stage by changing the scale or range of data from 0 to 1. Data normalization is necessary because microarray data has a significant range difference. The function of data normalization is presented by Eq. (1).

(1) $${y}^{\prime} = \displaystyle{{y - {y_{min}}} \over {{y_{max}} - {y_{min}}}}$$

where *y*′ is the value of features in the normalization domain, while the data’s value before the normalization process is y. At the same time, *y*_*min*_ and *y*_*max*_ refer to the smallest and largest values of all data in an attribute to be normalized, respectively. Algorithms of ML tend to be affected by noisy data. To avoid unnecessary complexity, noise should be decreased as much as possible in the inferred models (Karthik & Sudha, 2018; Daoud & Mayo, 2019; Adiwijaya et al., 2018). Two common types of noise can be known: (1) class noise and (2) attribute noise. Class noise is affected by samples classified as belonging to more than one class, which leads to wrong classifications. Simultaneously, the attribute causes attribute noise value errors, such as variables with wrongly measured and missing values.

**Figure 5:**
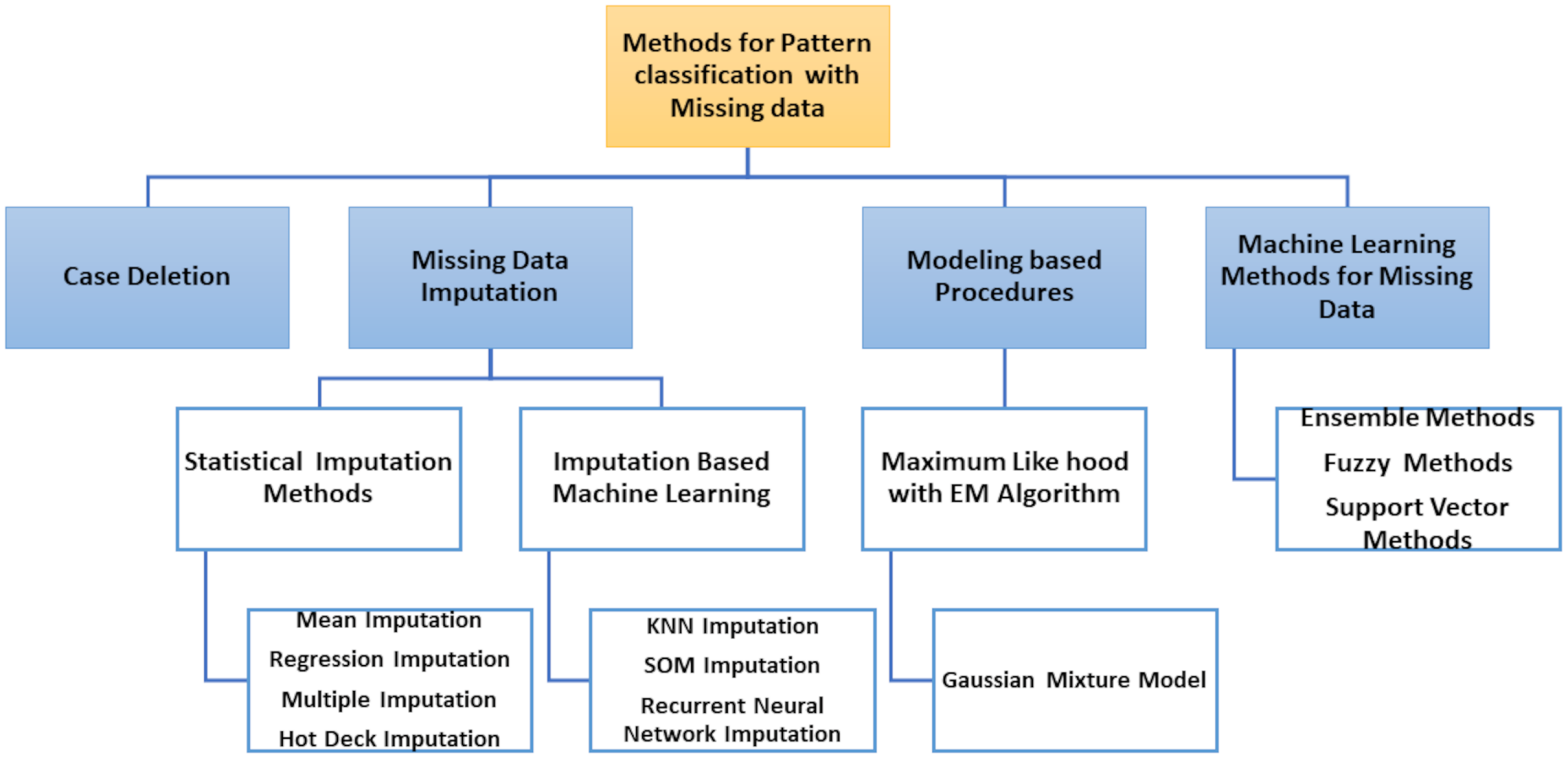
Methods for pattern classification with missing data.

The classification of patterns with missing data generally affects two issues: handling missing values and classifying patterns. The ability to handle missing data has become an essential requirement for pattern classification because inadequate data processing can lead to significant errors or false classification results. Most literature approaches can be grouped into four different types according to how both problems are resolved, as shown in Fig. 5.

1. Removal of incomplete cases and design of classifier using only the full data portion.
2. Imputation or estimation by editing set of missing data and the classification problem learning, *i.e*., with imputed values for complete data portion and incomplete patterns.
3. Use of model-based processes where the distribution of data is modelled using certain procedures, *e.g*. by expectation–maximization (EM) algorithm.
4. Use of ML methods in which missing values are included in the classification system.

The two first types of approaches are solved separately: the handling of missing value (data deletion, imputation) and the pattern classification, but the third type of approaches model the probability density function (PDF), used by the theory of decision in Bayes to classify the input data (complete and incomplete). Finally, the classifier is designed to process incomplete input data without previous estimates of missing data in the last types of approaches.

### Dimensionality reduction

As for increasing dimensions, the cost of computations also increases, usually exponentially. To get around this problem, it is essential to find a way to decrease the number of features in mind. Often two methods are used, which are feature selection and extraction (Daoud & Mayo, 2019; Adiwijaya et al., 2018).

#### Feature selection

Informative polymorphisms that are known as Tag SNPs and irrelevant ones are all found in the whole dataset. SNP data have high dimensionality, such as microarray gene expression, but it is larger several hundred times, which leads to the impractical of data analysis. Thus, removing the useless SNPs and extracting a small subset of those discriminants would help identify polymorphisms markers, which could be used as biomarkers. This process can be performed through feature selection, reducing a search space to facilitate classification tasks. Feature selection is divided mainly into the filter, wrapper, and embedded types. Before classification, task filtering techniques are applied to a dataset and usually to simple statistics, such as the t-statistic or F-stat, or the p-value that is used as the trait evaluation (Batnyam, Gantulga & Oh, 2013).

Conversely, wrapper methods take advantage of the learning algorithm to define a feature subset by integrating it within the feature search and selection. In the third type, the search for an ideal subset of features is included in the classifier’s construction and can search for the combined space of sub-feature sets and hypotheses. Table 4 provides a common classification of feature selection methods and shows most of the technique’s advantages and disadvantages (Batnyam, Gantulga & Oh, 2013, Van Rooij et al., 2019). An example of feature selection techniques is the information gain (IG) measure. It is dependent on the concept of entropy (Pereira et al., 2018; Joachims, 1998; Chandrashekar & Sahin, 2014; Khalid, Khalil & Nasreen, 2014; Kursa & Rudnicki, 2010). It is commonly used to measure feature suitability in filtering strategies that assess features’ individuality, and this method is quick. Suppose *D* (*A*_1_,*A*_2_,…,*A*_*n*_, *C*), *n* ≥ 1, be a dataset with *n* + 1 attributes, where C is the class attribute. Let m be the number of distinct class values. The class distribution of entropy in D, represented by Entropy(D), is defined by Eq. (2).

**Table 4:** The main feature selection techniques.

Feature selection	Advantages	Disadvantages
Filter	Univariate	
	Fast	Neglect dependencies with feature
	Scalable	Neglect the classifier interaction
	The selection of classifier is independent	
	Multivariate	
	Models feature dependencies	Slower
	Regardless of the classifier	Less scalable
	Better computational complexity than wrapper methods	Interaction is neglected with the classifier
	Deterministic	
Wrapper	Simple	Risk of overfitting
	Interacts with the classifier	More prone than randomized
	Feature of models is dependent	Algorithms to obtaining stuck in a local optimum (greedy search)
	Less computationally	The selection of classifier is dependent
	Intensive than randomized methods	
	Randomized	
	To local optima is less prone	Computationally intensive
	Models feature dependencies	The selection of classifier is dependent
		Overfitting with higher risk
		Than deterministic algorithms
Embedded	Interacts with the classifier	The selection of classifier is dependent
	Computational complexity is better than wrapper methods	
	Feature of models is dependent

(2) $$Entropy(D) = \sum\limits_{i = 1}^m {p_i}*\mathop {\log }\nolimits_2 *{p_i}$$

where *p*_*i*_ is the probability that an arbitrary instance of D belongs to the class *c*_*i*_. Eq. (2) defines the single-label strategy concepts, also known as IG attribute ranking (Mezlini & Goldenberg, 2017). It calculates the feature’s ability to distinguish between class values. In Daoud & Mayo (2019), For dealing with multi-label data, the C4.5 algorithm was adapted. This decision tree algorithm lets multiple labels at the tree leaves by calculating an entropy adaptation, as described by Eq. (3).

(3) $$Entropy.ML(D) = - \sum\limits_{i = 1}^l p({\lambda _i})*\mathop {\log }\nolimits_2 *p({\lambda _i}) + q({\lambda _i})*\mathop {\log }\nolimits_2 *q({\lambda _i})$$

where *p*(*λ*_*i*_) is the probability that an arbitrary instance in D belongs to the class label *λ*_*i*_, *q*(*λ*_*i*_) = 1 − *p*(*λ*_*i*_), and the number of labels is l in the dataset (Kursa & Rudnicki, 2010; Shaltout et al., 2015; How & Narayanan, 2004). They have adopted this formula to create an IG feature selection capable of dealing with multi-label data. By using IG as a filter approach, the feature selection can be performed with any multi-label classifier.

While embedded methods have the advantage of interacting with the classification algorithm, they have a lower computational cost than wrapper methods. It is referred to as the hybrid approach, which typically combines two or more feature selection algorithms of the various search strategies sequentially. For example, a less costly algorithm such as a filter might eliminate features first and use a more complex and costly algorithm afterward, such as a wrapper.

#### Feature extraction

Various techniques have been adopted to decrease the dimensions of gene data by choosing a subset of genes. There are several methods implemented to extract necessary information from microarrays and thus reduce their size. New variables are created by feature extraction as combinations of other variables to reduce the selected features’ dimensions. The feature extraction algorithms are consisting of two classes, which are linear and nonlinear techniques (Chu & Wang, 2005; Khodatars et al., 2020).

#### Linear

linear features extraction techniques suppose that the data is located in a low-dimensional linear subspace. The matrix factorization is projected onto this subspace. The most popular algorithm for dimensional reduction is principal component analysis (PCA). PCA finds main components in the data representing uncorrelated eigenvectors, representing a percentage of the variance of the data, using the covariance matrix and its eigenvalues and eigenvectors. PCA and many of its variants have been performed to reduce data dimensions in microarray data (Adiwijaya et al., 2018).

The steps for dimensional reduction algorithm using PCA, according to Chu & Wang (2005), are described below:

1. Let X be a matrix of input for PCA. X is training data consist of an n-vector with data dimension m.

2. For each dimension (X), calculate the mean data using Eq. (4).

(4) $$\bar X = \displaystyle{1 \over n}\sum\limits_{i = 1}^n {X_i}$$

where n = samples number or number of data observation, *X*_*i*_ = observation data.

3. The covariance matrix (CX) is calculated with Eq. (5), where (*X*) = mean data.

(5) $${C_x} = \displaystyle{1 \over {n - 1}}\sum\limits_{i = 1}^n ({x_i} - \bar x)({x_i} - \bar x{)^T}$$

4. The eigenvectors (*v*_*m*_) and eigenvalues (*λ*_*m*_ ) of the matrix of covariance is calculated using Eq. (6).

(6) $${c_x}{v_m} = {\lambda _m}{v_m}$$

5. In descending order, the sorting of eigenvalues.

6. The principal component (PC) is the eigenvector set corresponding to the eigenvalues sorted in step 5.

7. Based on the eigenvalues, the PC dimension will be reduced. Several ways are found for reducing. PC dimension based on eigenvalues, such as:

- Using a scree plot. The number of eigenvectors is determined based on the curve’s point, which is no longer sharply decreasing.
- Using the cumulative proportion of variance (eigenvalues) of the total variance (eigenvalues).

(7) $$PVV = \displaystyle{{{\lambda _i}} \over {\sum {\lambda _i}}} \times 100\%$$

Next, the number of eigenvectors was set by comparing the threshold with the cumulative proportion of variance. PC, which has reduced dimensionally ($\widehat {PC}$), is a matrix consisting of the largest k eigenvalues. Also, k eigenvectors are vectors corresponding to meet Eq. (8). The testing data (Y) dimensions are reduced by multiplying testing data with ($\widehat {PC}$) in Eq. (9).

(8) $$\displaystyle{{\sum\nolimits_{i = 1}^K {\lambda _i}} \over {\sum\nolimits_{i = 1}^n {\lambda _i}}} \times 100\% > Threshold$$

(9) $${y}^{\prime} = Y \times PC$$

It was argued that there is no guarantee that PCs are linked to the class variable when computing PCs of data sets. Supervised principal component analysis (SPCA) has therefore been introduced, in which the PCs based on the class variables were selected. This extra step is named the gene screening step. Even though the supervised PCA’s version works better than its unsupervised version, the PCA has a significant limitation: Nonlinear relationships, especially in complex biological systems, that often exist in data are not identified. SPCA works as follows (Hira & Gillies, 2015):

1. Calculate the relationship measurement between each gene using linear, logistic, or proportional hazard model results.
2. Using cross-validation of the models in step (1) select the most associated genes with the outcome.
3. Estimate PC scores using only the selected genes.
4. Using the model in step (1), fit regression with the outcome.

The method was extremely effective in determining essential genes, and in cross-validation tests, it was only outperformed by gene shaving, a statistical method for clustering, like hierarchical clustering. The term “shaving” is derived from the removal or having a percentage of the genes (normally 10%) that have the inner product with the smallest absolute with the leading PC. The main difference is that more than one cluster can be part of the genes.

#### Nonlinear

Reducing dimensions by using nonlinear techniques is applied by using many distinct ways. The low-dimensional surface can be mapped to high dimensional space to establish a nonlinear relation between the features. In theory, it is possible to use the lift function (x) to map the features over a space with higher dimensions. In a higher space, the relationship between the features can be shown as linear and easily discoverable. This is then sent to space with the lower dimensions, and their relevance can be considered nonlinear (Daoud & Mayo, 2019; Adiwijaya et al., 2018). Table 5 provides a comparison between techniques of feature selection and feature extraction.

**Table 5:** A comparison between selection and extraction techniques.

Method	Advantages	Disadvantages
Selection	Data Preserving for data interpretability	Discriminative power
		Lower times of training
		Reducing overfitting
Extraction	Higher distinguishing power	Interpretability of data is lost
	Overfitting Controlled when it is unsupervised	Switching can be costly

### Classification

After reducing the dimensional complexity of the data, the next step is the classification process. Classification is not only the primary purpose of this research, but also it is critical to detect biomarkers of complex diseases. At this stage, the data was diagnosed (classified) based on whether they are affected by a specific disease or not. Biomarkers that can detect and diagnose brain diseases accurately are urgently required. So, we introduce a comprehensive review of genome sequencing analysis for discovering complex genes related to genetic brain diseases. Most of the genetic variation is contributed by SNPs about the human genome. Many complex and common diseases are associated with SNPs like AD. Early diagnosis and treatment can improve by investigating SNP biomarkers at different loci of these diseases. The investigation of genetic variants in the human genome related to complex diseases is one of the most important study objectives (Sherif, Zayed & Fakhr, 2015; Saeys, Inza & Larranaga, 2007). So, we divided the review into three sections. Each section discusses a disease, which is summarized in Tables 8–10 and 12. Table 11 provides some of the available datasets that the researcher can use.

**Table 6:** The confusion matrix elements.

		Predicted	
		Normal	Abnormal
Actual	Normal	$TP$	$FN$
	Abnormal	$FP$	$TN$

**Table 7:** Some of the performance evaluation metrics.

Metrics	Description	Formula
Accuracy	This is a relation between the sum of TP and TN divided by the total sum of the population	$Accuracy = \displaystyle{{TP + TN} \over {TP + TN + FP + FN}}$
Sensitivity, Recall	This is a relation between TP divided by the total sum of TP, FN	$Recall = \displaystyle{{TP} \over {TP + FN}}$
Specificity	This is a relation between TN divided by the total sum of TN, FP	$Specificity = \displaystyle{{TN} \over {TN + FP}}$
AUROC	This metric is used to measure the average area under ROC	$TPR = \displaystyle{{TP} \over {TP + FN}}$ $FPR = \displaystyle{{FP} \over {FP + TN}}$
DSC	This is a relation between TP divided by the total sum of TP, FN, FP	$DSC = \displaystyle{{2*TP} \over {2*TP + FP + FN}}$
Precision	It is a relation between TP divided by the total sum of TP, FP	$Precision = \displaystyle{{TP} \over {TP + FP}}$
MCC	An effective solution overcoming the class imbalance issue comes from MCC	$MCC = \displaystyle{{TP*TN - FP*FN} \over {\sqrt {(TP + FP)(TP + FN)(TN + FP)(TN + FN} }}$

**Table 8:** An overview of ASD using different ML methods.

Authors	Application on diseases	Method	Results	Problem with method
Spencer et al. (2018)	ASD	FPM algorithms& contrast mining	Including 193 novel autism candidates as significant associations from connected 286 genes.	It is a challenge for FPM to store many combinations of items as a memory requirement problem.
Al-Diabat (2018)	ASD	Data mining and fuzzy rule	Fuzzy rules achieved accuracy up to 91.35% and 91.40% sensitivity rate.	Considering the assessment of features on the dataset in this study is not extensive and does not consider other target data sets like adults, adolescents, and infants.
Alzubi et al. (2017)	ASD	SVM, NB, linear discriminant analysis, and KNN	Classification accuracy up to 96%	Their work remains incomplete until the basis of genetic diseases, and traits well understand.
Krishnan et al. (2016)	ASD	ML approach	Area Under the Receiver–Operator curve (AUC) = 0.80.	The accuracy of the system needs to enhance
Cogill & Wang (2016)	ASD	SVM	A mean accuracy = 76.7%	-
Jiao et al. (2012)	ASD	DSs, ADTrees, and FlexTrees	DS and FlexTree With an accuracy = 67%,	One limitation of this work is that this study includes only 29 SNPs.

**Table 9:** An overview of cancer diseases using different ML methods.

Authors	Application on diseases	Method	Results	Problem with method
Ismaeel & Ablahad (2013)	Cancer disease	Classification by ANN	The best classifier with mean square error 0.0000001.	The system cannot apply as general for all diseases, so we need to propose a novel system to detect all diseases.
Jain, Jain & Jain (2018)	Cancer	NB classifier with cross-validation of stratified 10-fold	An accuracy up to 100%	In the microarray datasets, this approach is less reliable and having a small sample size.
Liu et al. (2008)	Periodontal Disease (PD), and Cardiovascular Disease (CVD)	NBSVM, and UDC.	The performance of NB and SVM better than (uncorrelated normal based quadratic Bayes classifier) UDC.	The number of features is limited, so the differences between the test’s accuracy levels were not noticeable.
Hussain et al. (2019)	Cancer	ANN, k-NN, DTs, NB, RF, and SVM	RF provides the best performance for the classification	

**Table 10:** An overview of AD diseases using different ML methods.

Authors	Application on diseases	Methods	Results	Problem with method
Tejeswinee, Shomona & Athilakshmi (2017)	AD	Classification techniques fed by each subset feature selection	Classification accuracy ${\rm \tilde 9}$3%	They cannot generalize all machine learning techniques are efficient in the classification of disease.
Park et al. (2018)	AD	Feature selection & ML &10-fold validation	Classification accuracy 91.6%	The system cannot apply as general for all diseases, so we need to propose a novel system to detect all diseases.
Abd El Hamid, Mabrouk & Omar (2019)	AD	Classification of ML techniques	Accuracy of NB 99.63%	Their system does not support integrating some metadata like gender, age, and smoking to check if these data are associated SNPs or not.
Shahbaz et al. (2019)	AD	ML & DM techniques	Accuracy of GLM is 88.24%	The model cannot be trained with unbalanced data and insufficient data for all classes of disease.
Zafeiris, Rutella & Ball (2018)	AD	ANN	The methodology can reliably generate novel markers	The power of the algorithms and speed are needed to be improved.
Bringas et al. (2020)	AD	CNN	The accuracy 90.91% and an F1-score of 0.897	This methodology needs to be used in a cloud architecture system to collect accelerometer data and a service that users can subscribe to for monitoring changes in the AD stage.

**Table 11:** Some publicly available datasets.

Authors	Application on diseases	Dataset
Al-Diabat (2018)	ASD	UCI data repository
Ismaeel & Ablahad (2013)	Breast Cancer disease	NCBI
Tejeswinee, Shomona & Athilakshmi (2017)	AD	(KEGG) database
Abd El Hamid, Mabrouk & Omar (2019)	AD	Phase 1 (ADNI-1)/Whole genome sequencing (WGS) datasets (Rémi et al., 2011).

**Table 12:** Different used applications to detect biomarkers for diseases.

Authors	Application on diseases	Methods	Results	Problem with method
Narayanan et al. (2019)	CAD for malaria, brain tumors, etc	ML	Their approach provided reasonable performance for all these applications	Their research needs to further extend by studying the ROI determined by class activation mapping.
Carter, Dubchak & Holbrook (2001)	Bacterial and archaeal	ML approach using ANN and SVM	RNA genes could be recognized with high confidence using the ML	Future studies are necessary to characterize the extent of these elements and the accuracy of predection.
Narayanan, Hardie & Kebede (2018)	Lung cancer	SVM	CAD perform sensitivity with 82.82	They should optimize the feature set for SVM classification.
Yang et al. (2020)	AD	ML	The AUC to ${\rm \tilde 0}$.84	
De Velasco Oriol et al. (2019)	LOAD	ML	Classification performance is 72%	The classification performance needs more enhancement.
Ahn et al. (2019)	Business big data business analytics	Fuzzy logic dependent on ML tools	Their improved version led to benefits of additional 10% accuracy.	Their tool need to handle further outliers and edge cases and explore factors that are more explicit and/or implicit in business processes.
Gao & Tembine (2016)	Network systems	Distributed Mean-Field	Mean Square Error =2.01	They use no more complex detectors based on vision. In addition to reducing measuring noise they don’t use deep learned features.
Xu et al. (2020)	Smoking prediction Models	SVM and RF	AUC of SVM = 0.720, and AUC of RF = 0.667.	Their work in the future needs to identify the inner complex relations between these SNPs and smoking status.

#### Support vector machine (SVM)

SVM is considered the well-known classification technique, which can be used in disease diagnosis. SVM is depend on statistical learning theory (Hira & Gillies, 2015; Tang, Suganthan & Yao, 2006; Bansal et al., 2018). It is an algorithm that identifies a specific linear model, which maximizes the hyper-plane margin. Maximizing the margin of hyperplanes will maximize classes separation (Abd El Hamid, Omar & Mabrouk, 2016; Breiman, 2001). The training points closest to the margin of the cloud are the support vectors. These vectors (points) are only used to define the boundaries between classes. Assuming the classes are linearly separable, they obtain the hyper-planes with maximum margin to separate them (Chu & Wang, 2005; El-Gamal et al., 2018; Gayathri, Sumathi & Santhanam, 2013; Karthik & Sudha, 2018; Farhadian, Shokouhi & Torkzaban, 2020).

Quadratic Programming (QP) methods are well Known methods for solving constrained problems to determine the optimum line for the data. When the data cannot be separated linearly, the data is mapped to a larger dimensional space using a kernel function to be divided linearly in this new space. Different kernel functions, like linear, radial, polynomial and sigmoid, can be used in this situation. The optimal hyperplane is the initial goal that should be found. The hyperplane is the border between classes. Not only separating between classes is the primary goal of the optimal hyperplane, but it also increases the margin. Margin is the longest distance between the hyperplane and the closest data (support vector) in each category (Chu & Wang, 2005; Karthik & Sudha, 2018; Farhadian, Shokouhi & Torkzaban, 2020).

1. Let {*x*_1_,*x*_2_,…,*x*_*n*_ } is a dataset of real value. It is a dataset of real value.

2. Assume classes is *y*_*i*_ ∈ { − 1,1} is the label of data, w is a weighted vector. Eq. (10) can be written as follow to estimate hyperplane:

(10) $$f(x) = w{x_i} + b = 0$$

Then, from Eqs. (10)–(12) are obtained:

(11) $$w{x_i} + b \ge + 1\ for\;class + 1$$

(12) $$w{x_i} + b \le - 1\ for\;class - 1$$

where x is the input data, w is the normal plane, and b is the position relative to the middle field coordinates.

3. The main goal of SVM is to find high levels between two classes that increase margins.

(13) $$mi{n_w}\displaystyle{1 \over 2}{\rm \parallel }w{{\rm \parallel }^2}$$

(14) $${y_i}(w{x_i} + b) - 1 \ge 0$$

The problem can be solved in quadratic programming using the Lagrange multipliers shown in Eq. (15):

(15) $$L(w,b,\alpha ) = \displaystyle{1 \over 2}{\rm \parallel }w{{\rm \parallel }^2} - \sum\limits_{i = 1}^I {\alpha _i}({y_i}((w{x_i} + b) - 1))$$

(16) $$L(\alpha ) = \sum\limits_{i = 1}^I {\alpha _i}\displaystyle{1 \over 2}\sum\limits_{j = 1}^I {\alpha _i}{\alpha _j}{y_i}{y_j}{x_i}{x_j}$$

where, *α*_*i*_ is the weight (parameter obtained from the Lagrangian Multipliers).

4. For making decisions, Eq. (17) is used for linear equations, while Eq. (18) is used for nonlinear equations:

(17) $$f({x_d}) = sign\bigg(\sum\limits_{i = 1}^n {\alpha _i}{y_i}({x_i},{x_d}) + b\bigg)$$

(18) $$f({x_d}) = sign\bigg(\sum\limits_{i = 1}^n {\alpha _i}{y_i}K({x_i},{x_d}) + b\bigg)$$

where n is the number of support vectors and *x*_*d*_ is the test data and *K*(*x*_*i*_,*x*_*d*_) is the kernel function used, as per Eq. (19).

Linear Kernel:

(19) $$K({\vec x_i},{\vec x_d}) = ({\vec x_i},{\vec x_d})$$

The radial basis function kernel (RBF) is shown in Eq. (20):

(20) $$K({\vec x_i},{\vec x_d}) = exp\bigg(\displaystyle{{ - |{{\vec x}_i} - {{\vec x}_d}{|^2}} \over {2{\sigma ^2}}}\bigg)$$

#### K-nearest neighbors algorithm (KNN)

KNN is considered one of the most straightforward techniques of DM. It is also known as a memory-based classification because it is at run-time needed for the training examples to be in the memory (Shouman, Turner & Stocker, 2012; Alpaydin, 1997). However, the major drawback of the KNN classifier is the large memory requirements, which are needed to store the entire sample. When the sample is large, the response time on a serial computer is also significant. In the first step, the closest point to P is found, where P is the point for which the label needs to be predicted. Then, the label is assigned to P at the closest point. Second, the k nearest to P is identified, and the majority of its k neighbors classify points by vote. The most voting class of each object is predicted by its class and by the most votes class. The distances between these points, such as Euclidean, Hamming, Manhattan, and Minkowski, are calculated using distance measures for finding the nearest similar points. The algorithm has the following basic steps: distance calculation, find closest neighbors, and labels voting.

In order to calculate the distance between P and its closest neighbors, there are three most commonly used distances measures:

1. The difference between features is calculated using the Euclidean distance when dealing with continuous features. If the first instance is (*a*_1_, *a*_2_, *a*_3_,…, *a*_*n*_) and the second instance is (*b*_1_, *b*_2_, *b*_3_,…,*b*_*n*_), by the following formula the distance between them is computed :

(21) $$EuclideanDistance = \sqrt {{{({a_1} - {b_1})}^2} + {{({a_2} - {b_2})}^2} + \ldots {{({a_n} - {b_n})}^2}}$$

The main problem is that the frequencies of large values swamp into small values in dealing with the Euclidean distance formula.

2. Manhattan distance: It is the distance that is usually preferred over the more common Euclidean distance where data are of a high dimension.

(22) $$Manhattandistance = \sum\limits_{i = 1}^K |{a_i} - {b_i}|$$

3. Minkowski distance:

(23) $$Minkowskidistance = \bigg(\sum\limits_{i = 1}^K {({a_i} - {b_i})^q}{\bigg)^1}/q$$

#### Logistic regression (LR)

LR is one of the ML classification algorithms for analyzing the dataset in which there are one or more independent variables that identify the outcome and the categorical dependent variable (Bertram, Lill & Tanzi, 2010). In many ways, LR is the natural complement of normal linear regression when the target variable is categorized (Sa’id et al., 2020). For output (dependent) variable Y to classify two class and input (independent) variable X, let *g*(*x*) = *pr*(*X* = *x*) = 1 − *pr*(*X* = *x*), the LR model has a linear form for logit probability as follows:

(24) $$logit[g(x)] = log\bigg(\displaystyle{{g(x)} \over {1 - g(x)}}\bigg) = \alpha + \beta x$$

where $({{g(x)} \over {1 - g(x)}})$ is called odd. The logit has a form of linear approximation. It is equated with the logarithm of the odds. The parameter *β* is the rate of increase or decrease of the S-shaped curve of g(x).

### Performance evaluation metrics

Performance evaluation metrics are used to measure the classification model’s performance and investigate how a model works well to achieve the goal. On the test dataset, performance evaluation metrics are used to estimate the classification model’s performance and effectiveness, chosen correct metrics. It is essential, such as the confusion matrix in Table 6 to evaluate the model performance. Some commonly used performance metrics include accuracy, precision, and Matthews’s correlation coefficient (MCC). which are listed in Table 7 (Jain, Jain & Jain, 2018; Singh & Sivabalakrishnan, 2015; Raj & Masood, 2020; Le et al., 2020; Do & Le, 2019).

## Literature Review on Complex Disease & Applications

### Autism spectrum disorder (ASD)

**Figure 6:**
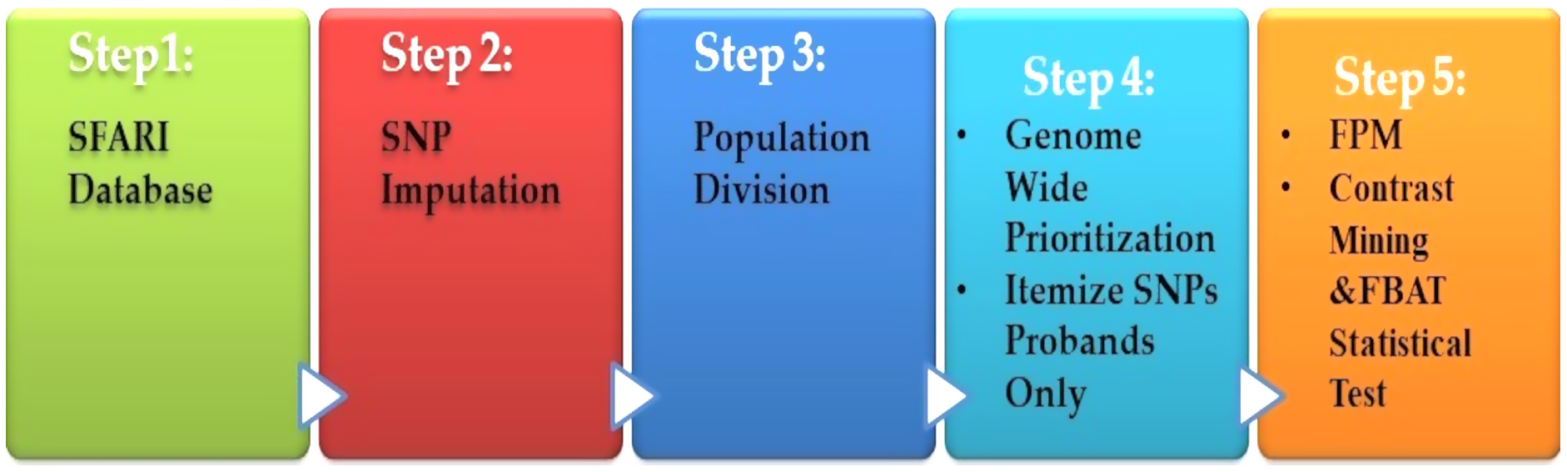
Diagnosis system for ASD based on DM techniques.

Spencer et al. (2018) presented a heritable genotype system. Their system consisted of five stages, as shown in Fig. 6. The preprocessing is the first step, which contained the missing value imputations for the SNPs, and then they selected the most SNPs significantly. The second stage is population division. Prioritization is used as a primary association for each genome-wide subgroup procedure. The third stage is the prioritization of genome-wide by turning to the question: what sets of SNPs are they to test? The answer is to use the minimum threshold support in the frequent models (FPM) algorithm. The filter is performed for SNP sets, according to its prevalence among the affected population. The fourth stage is FPM, one of the most DM techniques that excellently identifies the most common occurrence in feature combinations. The data must be interpreted in binary form with both reference states in one person. The presence or absence of the item in FPM shows potential interactions among variants. Finally, the stage is the UICsup, which is a contrast mining utilizing. In the end, the comparison with another application is carried out, and its system for all was superior.

Al-Diabat (2018) presented an autism screening process which comprises presenting some questions to parents, family members, and caregivers to answer the child’s behalf to identify potential autism traits. The data mining and fuzzy rule are adapted to improve the screen efficiency and accuracy, which is considered one potential solution. To discover the features of ASD, the fuzzy unordered rule induction (FURIA) algorithm was evaluated. The automated method, FURIA, is designed to examine patterns from controls and historical cases. After then, they used the models to determine the possibility of autism traits in new individuals. The higher performance of fuzzy data mining models is evidenced by experimental results regarding predictive accuracy and sensitivity rates.

Alzubi et al. (2017) developed an accurate method based on hybrid feature selection for identifying the most SNPs that are informative and chosen an optimal SNP subset. Their method was based on the fusion of the wrapper and filter method. The system performance was evaluated against four feature selection methods and four classifiers on SNPs’ five different data sets. The experiment results show the adopted feature selection approach’s efficiency and achieve classification accuracy up to 96% for the used data set. Overall, they concluded that the whole genome could efficiently differentiate between people with complex diseases and healthy individuals. Their method has been validated in an independent and large case and evidence study.

Krishnan et al. (2016) introduced a complementary ML approach based on a network for the human brain’s specific genes to present a wide prediction genome of autism risk genes, comprising candidates who may be hundreds is minimal or no previous genetic evidence. Leveraging these genome- and network-specific predictions of the brain, we showed that a large group of ASD genes converge in fewer major pathways and developmental stages of the brain. Finally, they identified potentially pathogenic genes within autism-related CNVs and suggested genes and pathways that are likely mediators of ASD across multiple CNVs.

The model decision function output ranks the gene lists comprising an ASD risk gene and adjacent genes. Cogill & Wang (2016) developed SVM with training on brain developmental gene expression data for classification and prioritization of ASD risk genes. The pre-reduction process has been shown to improve the accuracy of the SVM classification. The results showed that each filter procedure identifies different gene sets with a specific gene repetition. Due to the high variance in ASD of several genes, further selection steps are necessary.

Jiao et al. (2012) presented an SNP-based predictive model which could predict the severity of ASD’s symptoms. They divided 118 ASD children into a moderate group of autisms (n = 65) and a severe group of autisms (*n* = 53). 29 SNPs of 9 ASD-related genes were obtained for every child. They applied three ML techniques to create predictive models: Decision stumps (DSs), ADTrees, and FlexTrees. With an accuracy = 67%, DS and FlexTree produced modestly better classifiers. All models of the SNP rs878960 in GABRB3 were selected and linked to the CARS evaluation.

### Cancer & brain diseases

**Figure 7:**
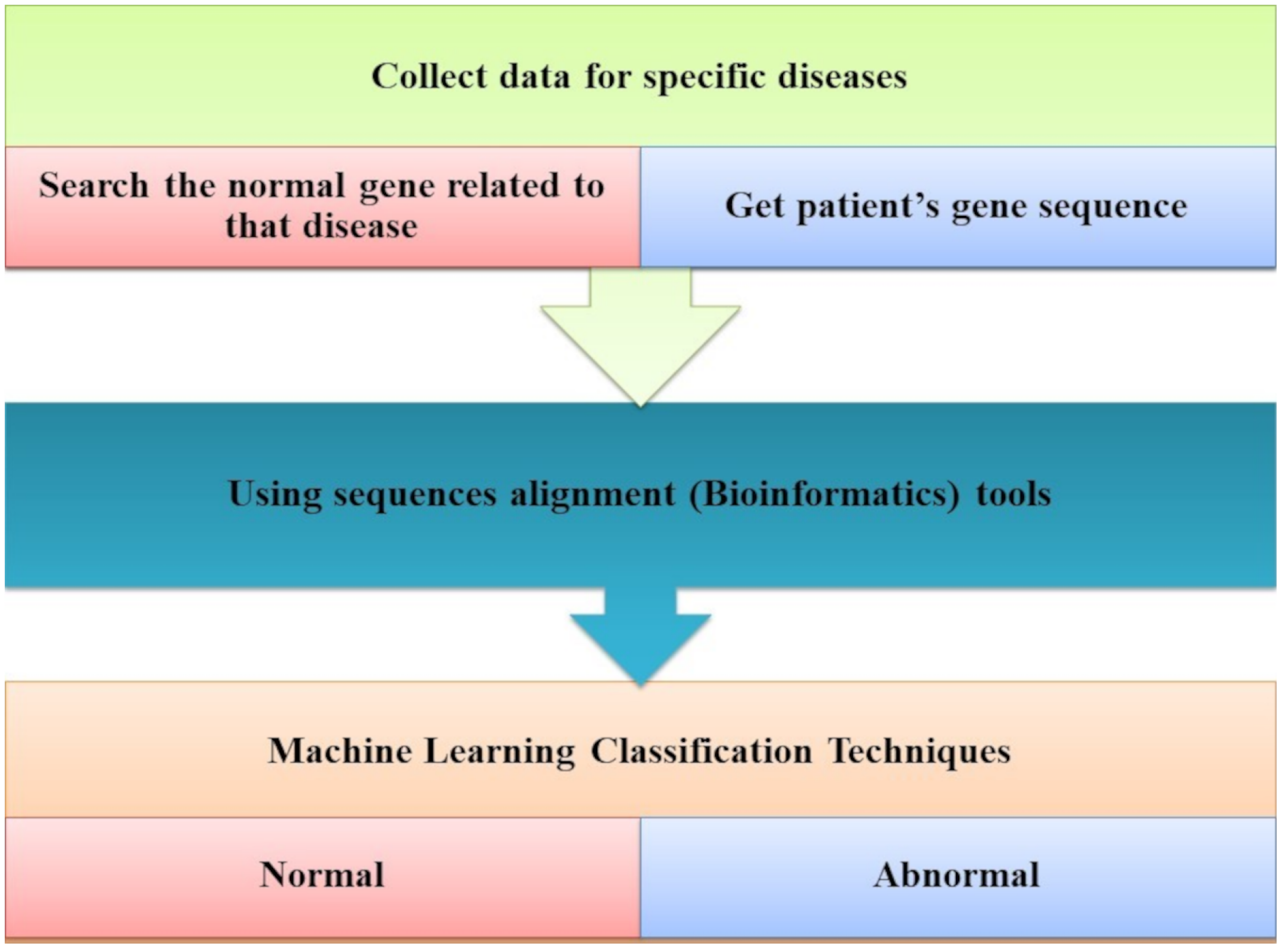
The main tasks of the gene-based analysis system.

Ismaeel & Ablahad (2013) developed a method that can predict the disease by mutations. Bioinformatics techniques were used to train and used back-propagation algorithms to test whether the patient has the disease or not on the collected data, using all expected mutations for genes of some diseases (*e.g*., BRCA1 and BRCA2). They Implemented their method as the first way of predicting the disease based on mutations in the gene sequence causing this disease which showed two decisions were achieved successfully, as shown in Fig. 7. The first way was to diagnose whether a patient had cancer mutations or not by using bioinformatics techniques. Back-propagation is the second classification of these mutations.

Jain, Jain & Jain (2018) introduced a two-phase hybrid model for cancer classification. A low-dimensional set is selected for this model of prognostic genes. For biological samples classification into binary and multi-class cancers using naïve Bayes (NB) classifier with a cross-validation stratified 10-fold. Different cancer types have been evaluated in their system by using 11 microarray datasets. The experiment results are compared with seven methods. Their system achieved better results, showing the classification accuracy and the selected number of genes in most cases. It is obtained classification accuracy for seven datasets up to 100% with prognostic gene subset with very small sizes (up to <1.5%) for all datasets.

Liu et al. (2008) developed a supervised recursive feature addition (SRFA) method for feature selection. This approach chose candidate features/SNPs. Supervised learning and statistical measures are combined to control the redundancy information and enhance classification performance in association studies. Additionally, they have developed a support vector-based recursive feature addition (SVRFA) scheme in the SNP association analysis of disease.

Hussain et al. (2019) analyzed the DNA sequences of cancer patients using various classification techniques. Based on this study, it can be concluded that people’s cancer diseases can be diagnosed easily based on their DNA sequences. Different classifiers have been used to analyze purposes, such as an artificial neural network (ANN), KNN, decision trees (DTs), and fuzzy classifiers, NB, random forest (RF), and SVM. Based on the analysis of results, it has been found that these classifiers provide sufficient performance in terms of accuracy, recall, specificity, and other parameters. By using the gene dataset, the paper used different classifiers to analyze the data. They found the RF provides the best performance for the classification of cancer patients.

### Alzheimer’s disease (AD)

**Figure 8:**
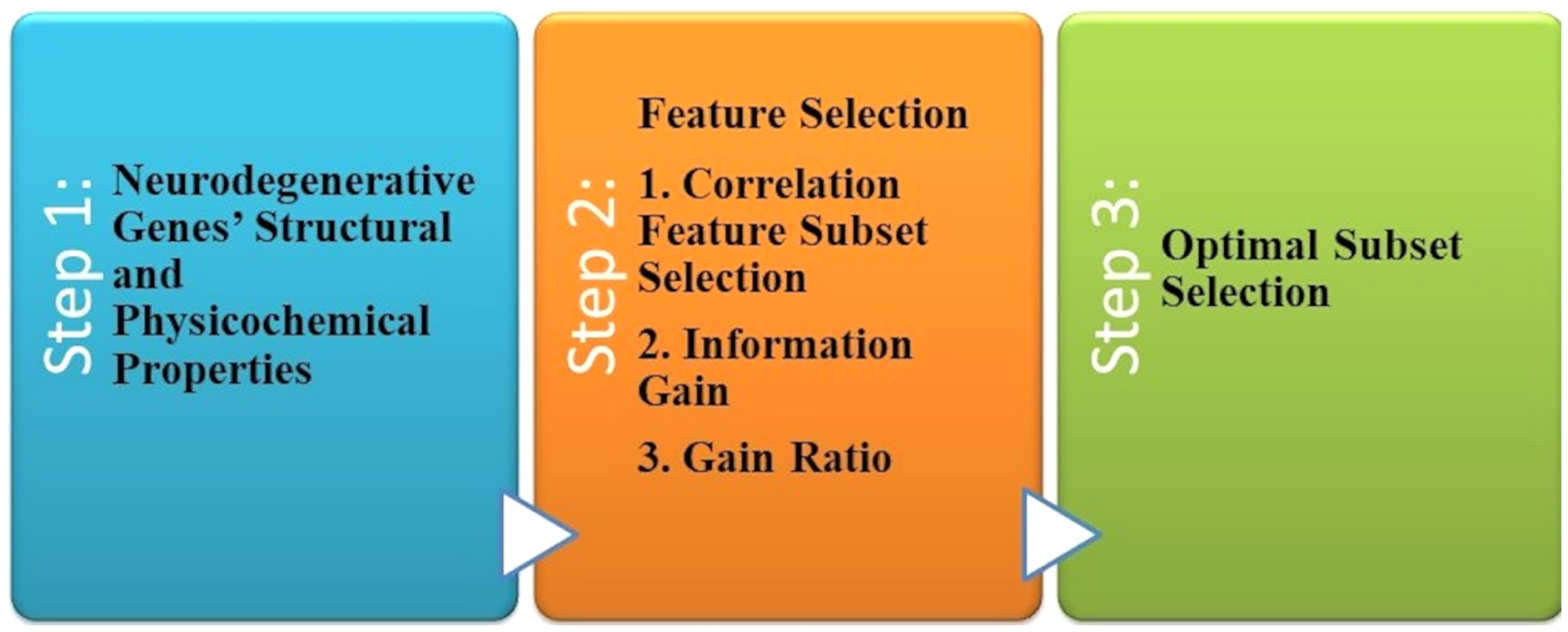
The computational system for AD disorders.

The most common forms of dementia that degenerate neurons are AD and PD found in the brain cells. Tejeswinee, Shomona & Athilakshmi (2017) described a computational framework to investigate neurodegenerative disorders, as shown in Fig. 5. The collection of data that they performed has not been the computational explorations of earlier utilization. In the first stage, the genes related to AD and PD are collected as the dataset generation. Genes uniquely of AD are 74 and 38 genes uniquely of PD, representing 112 of genes collected. Both diseases have 95 common genes. Feature selection is the second step. Three feature selection methodologies are used in this study to select the optimal feature set. The result revealed that it is the best-optimized feature subset. This subset is used to feed the six classification algorithms individually to each of them in the classification phase, as shown in Fig. 8. The accuracy of all these algorithms was measured when predicting the correct diagnostic class.

**Figure 9:**
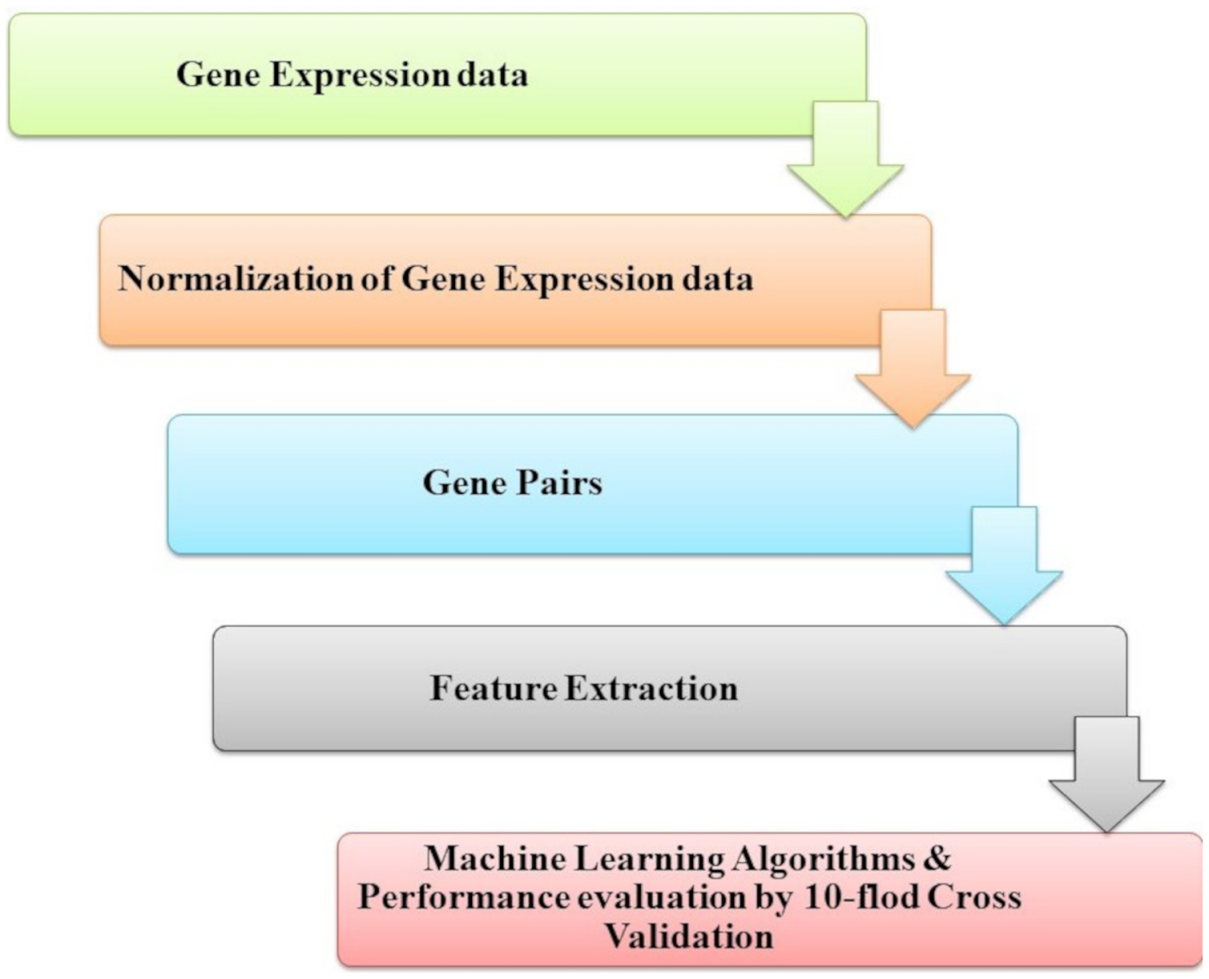
The identification of genetic interactions system.

Park et al. (2018) introduced genetic interactions based on ML from heterogeneous gene expression profiles, as presented in Fig. 9. Related gene determination of disease and dis-ease mechanisms is a research goal and necessary process; this problem has been approached with many studies problems by analyzing gene expression profiles and datasets interaction for genetic networks. Among pairs of genes, correlations or associations must be determined to construct a gene network. However, when heterogeneous data for gene expression is noisy with high levels for assigned samples to the same condition, it is difficult to specify whether a gene’s pair represents a significant (GGI). To find a solution for this challenge, they introduced an RF-based method to classify data of gene expression of significant GGIs. The model is trained by defining sets of novel features and utilizing various datasets with high confidence interactome.

Abd El Hamid, Mabrouk & Omar (2019) developed a method for detecting biomarkers SNPs associated with the disease with high accuracy classification, leading to early disease prediction and diagnosis. For discovering new biomarkers, ML techniques are utilized for the dis-ease. Many common diseases have SNPs related to AD. SNPs are recognized for this disease as significant biomarkers. In its early stages, they allow in understanding and detecting the disease. They apply many techniques that are performed on all genetic data of AD. They achieved the highest accuracy of classification. The results detected that K2 learning and NB algorithms accomplished an accuracy of 98.40% and 98%, respectively.

Shahbaz et al. (2019) introduced an AD classification using ML techniques to classify AD. In their paper, they performed six different ML and DM algorithms. The first step in their work is a data processing and feature selection. The second step is to partition data partitioning using 70% of training data and the reset for testing. Finally, classification techniques are used. Five stages of AD’s can efficiently classify using the generalized linear model (GLM) described in the study results on the test dataset with 88.24% accuracy. In medicine and healthcare, these techniques can be successful for early disease detection and diagnosis.

Zafeiris, Rutella & Ball (2018) discussed, explored, and evaluated an integrated ANN pipeline in AD for biomarker discovery and validation. Over time, the most popular form of dementia and no specific cause and no treatment available. The developed system consists of analyzing data that are public with a categorical and predicting gene interactions. A continuous stepwise algorithm is used and further examined through network inference. Novel markers can be generated by their methodology and another well-known study that could be used to guide future research in AD.

Bringas et al. (2020) implemented deep learning model AD stage identification. Data from a daycare center collected for one week from 35 AD patients on smartphones were used. The data sequences for each patient recorded changes in the accelerometer during daily operations and labeled with the disease stage (early, middle or late). Their methodology uses the CNN model to identify the patterns which identify each step. Their methodology is used to process these time series. As a result, the 90.91% accuracy and an F1-score of 0.897 were achieved through the CNN-based method, which greatly improves the results achieved by classifying features.

### Different applications

Narayanan et al. (2019) provided a computer-aided detection (CAD), and it is considered as a hot research area that is attracting significant interest in the past ten years. ML algorithms have been widely used for this application because they provide clinicians with valuable opinions to make the right decision. Regardless of the many ML models available for medical imaging applications, not much has been implemented in the real world due to the inexplicable nature of the network’s decisions. Their research paper investigated the results supported by deep neural networks to detect malaria, brain tumors, etc., in various imaging methods. They visualized category activation mappings for all applications to improve understanding of these networks. This work will help data science experts understand their models and assist clinicians in the decision-making process. The main drawback of their work should extend by studying the region of interest (RoI).

Carter, Dubchak & Holbrook (2001) developed an ML approach using ANN and SVM to extract standard features among known Ribonucleic acids (RNAs) to predict new RNA genes in regions not shown on them. Results showed RNA genetic differences in composition and structural parameters in known RNAs compared to non-coding sequences could be recognized with high confidence using M L-Approach in both bacterial and archaeal genomes. Besides cross-validation testing, highly predicted sequences in *E. coil*, M.geneitalium, etc., not involved in the training datasets, were experimentally distinguished and reported in the literature as expressed fRNAs.

Narayanan, Hardie & Kebede (2018) presented a study of lung cancer and explored SVM’s performance based on a wide range of features. The SVM performance was studied according to the number of features. Their results demonstrated greater robustness, faster computation with a wide range of features, and less prone to overtraining than conventional classifiers. Besides, they also offered a computationally efficient approach to select SVM features. Results are presented to the publicly available 2016 lung nodule analysis dataset. Their results show that the SVM is superior to the Fisher linear discrimination classifier based on 10-fold cross-validation by 14.8%.

Yang et al. (2020) provided a review that introduced sequencing technology development and explains the structure of DNA sequence data and sequence similarity. Second, they analyzed the necessary DM process, summarized several of the significant ML algorithms, and highlighted the future challenges faced by ML algorithms in extracting biological sequence data and possible future solutions. Third, they reviewed four typical applications of ML in DNA sequencing data. Fourth, they analyzed the background and relevance of the corresponding biological application. Fifth, they have systematically summarized the evolution and potential problems in the field of DM for DNA sequencing in recent years. Finally, they summarized the content of the review and looked forward to the future for some research directions.

Bellinger et al. (2017) performed a systematic review of the application of DM and ML methods in the epidemiology of air pollution. A systematic review identified current trends and challenges and explored recent trends for applying data extraction methods to air pollution epidemiology. Their work presented that DM is increasingly performed in the epidemiology of air pollution. The potential is to enhance air pollution epidemiology continues to grow with DM’s advances for temporal and geospatial mining and deep learning. New sensors and storage media, which allow greater and better information, also support this. This shows that a large number of successful applications can in the future be expected.

Bracher-Smith, Crawford & Escott-Price (2021) introduced a review for psychiatric disorders based on ML for genetic prediction. ML methods have been performed to make predictions in psychiatry from genotypes, with the potential to enhance the process of prediction of outcomes in psychiatric genetics; however, their current performance is unclear. This paper aimed to systematically review ML methods for psychiatric disorders prediction from genetics alone and estimate their discrimination, bias, and implementation.

Romero-Rosales et al. (2020) compared three ML methods that have been shown to build robust predictive models (genetic algorithms, LASSO, and progressive wisdom) and provided the involvement of markers from misclassified samples to enhance the accuracy of overall prediction. Their results described that adding markers from a prototype plus model markers fitted to misclassified samples enhances the AUC about 5%, to ≈ 0.84, which is very competitive using only the genetic information. The computational strategy applied here can support good ways to enhance classification models for AD. Their work could have a positive effect on early AD diagnosis.

De Velasco Oriol et al. (2019) performed systematic comparisons of representative ML models to predict the late onset of AD (LOAD) from the ADNI cohort’s genetic variance data. Their experimental results demonstrated that classification performance is the best-tested model resulting in 72% of the area under the receiver operating characteristic (ROC) curve.

Mishra & Li (2020) introduced the first intensive reviews for applying AI technology in medicine and the current genetic research state in AD. Next, the extensive review concentrated on applying AI in genetic research of AD, including diagnosis and prediction of AD based on genetic data, analysis of genetic variance, gene expression profile, and gene expression. AD analysis based on the knowledge base. Although several studies have yielded some meaningful results, it is still in the preliminary stage. Fundamental shortcomings include database limitations, failure to leverage AI to conduct a systematic biological analysis of multi-level databases, and a theoretical framework for analysis results. Finally, the future direction of development is to aspire to high quality, comprehensive sample size, and data sharing resources should be developed. An AI analysis strategy for multi-level system biology is a development trend. Computational innovation may play a role in constructing and validating the theoretical model and designing new intervention protocols for AD.

Ahn et al. (2019) introduced fuzzy logic dependent on ML tools for enhancing business big data business analytics in complex AI environments. Specifically, they have provided a suitable and extended the traditional C4.5 algorithm by incorporating fuzzy logic for business processes. To classify and predict the individual wage, government staff, help take actions appropriate to those in need and/or make better use of their available resources. The resulting tool supported analysts in this process. Additionally, they have also released an enhanced version, and this base copy has been reinforced by branch extension. The enhanced version speeded up the mining process and the business analytics and led to higher accuracy.

Gao & Tembine (2016) presented networked systems for the effective way of extraction and utilization and the availability of accurate prediction, proactive tools of mean-field type dynamical systems. They introduced large-scale networked systems based on the distributed mean-field filter (DMF). The filter exploits the network’s topology and breaks it down into highly independent components concerning marginal mean-field correlations. The experiments showed for two object tracking scenarios are performed to illustrate the performance of their algorithm. Results of the evaluation show that DMF superior to the existing filtering algorithms.

Yazdani et al. (2020) introduced simultaneously complex and common features that arise from multiple genes interacted and regulated. Therefore, to unravel the fundamental biological networks, it is vital to characterize genes’ interconnectivity. They have systematically combined transcription, genotyping, and Hi-C data to determine the interconnections between individual genes as a causal network. They used various ML techniques to extract information from the network, determine differential regulatory patterns between cases, and control schizophrenia.

Yazdani et al. (2018) introduced a method which is known as Bounded Fuzzy Possibilistic Method (BFPM) that takes into account preserving the flexibility of the search space for higher accuracy cluster sampling (Yazdani, 2020). The method assessed samples for their movement from one cluster to another. This technique lets us finding important samples in advance of those with the potential to belong to other clusters in the near future. BFPM was performed on the metabolism of individuals in a lung cancer case-control study. Metabolism as close molecular signals of actual disease processes may be powerful biomarkers of the current disease process. They aimed to find out whether healthy human serum metabolites can be distinguished from those with lung cancer. With BFPM, some differences were noticed, pathology data were evaluated, and essential samples were identified.

Xu et al. (2020) used the methods of SVM and RF for developing smoking prediction models. For a model building of 10- fold cross-validation, they first used 1,431 smokers and 1.503 non-smokers and tested the model prediction models on independent datasets of 213 smokers and 224 non-smokers. AUC of 0.691, 0.721 and 0.720 for training, tests, and independent test samples were obtained by SVM with the 500 top SNPs, selected using LR methods (*p* < 0.01). AUCs 0.671.665 and 0.667 were obtained for the training, the tests, and independent test samples of 500 top SNPs selected using LR (*p* < 0.01).

Le & Nguyen (2019) applied DL in the prediction of SNARE proteins, which is one of life science’s most essential molecular functions. Several human illnesses involve a functional loss of SNARE proteins (*e.g*., neurodegenerative, mental illness, cancer, and so on). There is thus a critical problem in understanding these diseases and designing the drug targets by establishing a precise model to determine their functions. With 2D convolutional neural network (CNN) and position-specific scoring matrix profiles, their SNARE-CNN model could identify the accuracy reached with the SNARE proteins of 89.7%.

In many aspects of cellular life activities, antioxidant proteins are essential. Cell and DNA are protected against oxidative agents. In this study, Ho Thanh Lam et al. (2020) developed an ML model from a benchmark set of sequence data that was applied for this prediction purpose. The experiments have been performed through 10-fold cross-validation during the training and validated by three separate datasets. On the optimum set of sequence features, various ML and DL algorithms were evaluated. Among them, RF was identified as the best model for identifying antioxidant proteins with the highest performance. Their optimal model has achieved 84.6% high accuracy.

## Critical Discussion and Future Challenges

Microarray data’s emergence poses many ML research challenges due to its large dimensional nature with small sample size. Aside from the notable disadvantage of having many features of a small number of samples, researchers also have to meet the unbalanced classes characterizing the data, testing datasets and extracting training in various cases, and the presence of outliers (dataset shift). For all these reasons, new technologies continue to emerge every year, aiming to improve the classification accuracy of previous approaches and help biologists discover and understand the underlying mechanism linking gene expression to diseases. So, we added Table 13, which displays the classification accuracy of previous approaches.

**Table 13:** Different applications of ML and their accuracy.

Authors	Methods	Accuracy	Disease
Alzubi et al. (2017)	ML	96%	ASD
Tejeswinee, Shomona & Athilakshmi (2017)	ML	93%	AD
Al-Diabat (2018)	DM and Fuzzy Rules	91.35%	ASD
Jain, Jain & Jain (2018)	NB	100%	Cancer
Park et al. (2018)	ML	91.6%	AD
Narayanan, Hardie & Kebede (2018)	SVM	82.82%	Lung Cancer
Abd El Hamid, Mabrouk & Omar (2019)	NB	99.63%	AD
Shahbaz et al. (2019)	GLM	88.24%	AD
Bringas et al. (2020)	CNN	90.91%	AD
Yang et al. (2020)	ML	84%	AD

So, it is useful in biological research to determine individuals’ phenotype and genotype characteristics. The phenotype connected with physical appearance, and the genotype is the genotype of the individual. SNPs make the individual different from others as well as help in determining genetic variation in the population. The main factor in identifying the relationship of an individual to a population is genomic variation. SNPs play an essential role in genome-based disease detection, drug design, drug and isolation reaction to an environmental factor, such as toxins and disease development risk in a population, so it is used for this purpose (Tahir & Sardaraz, 2020).

**Figure 10:**
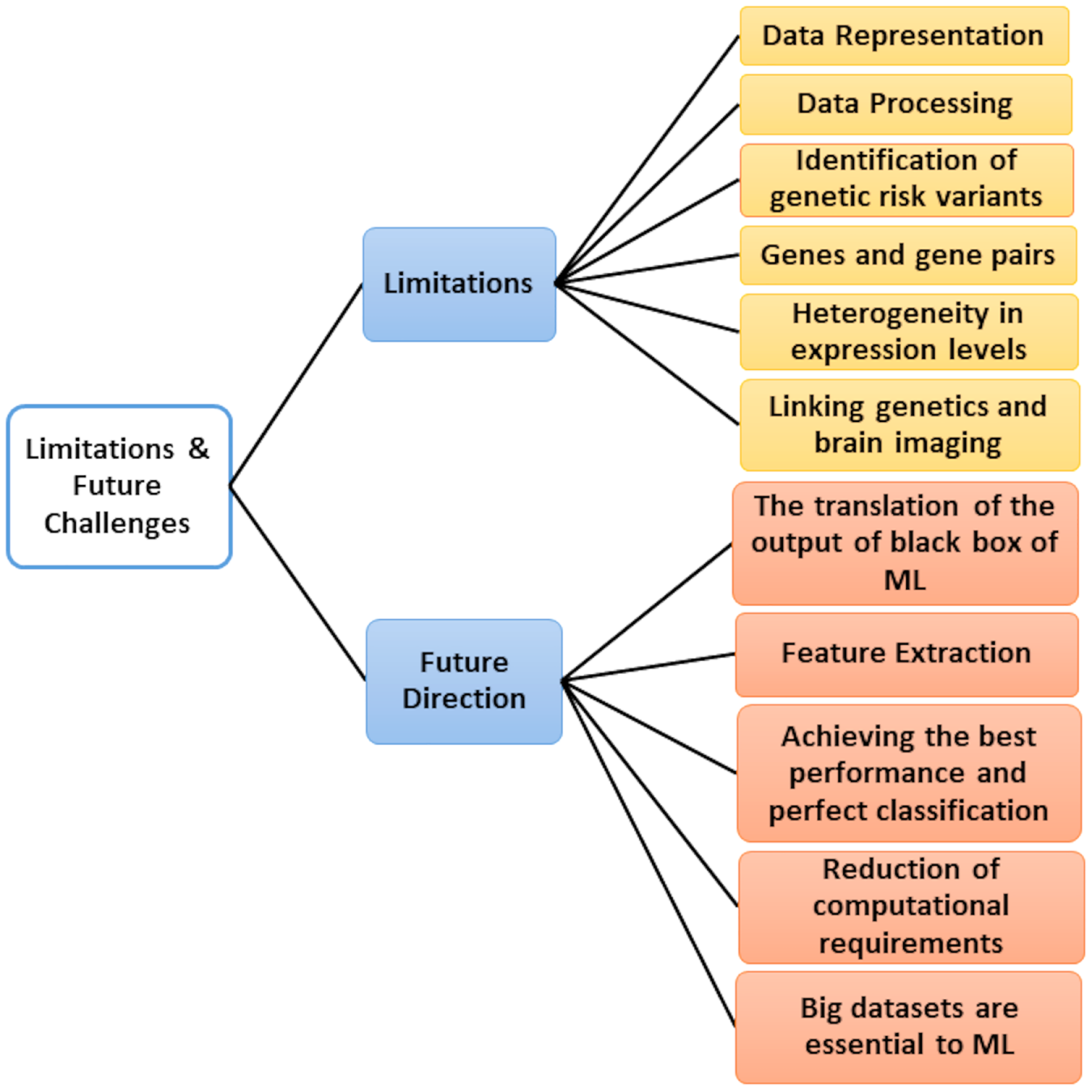
Limitations and future directions.

Biomarkers are urgently needed for the early detection of complex genetically related brain diseases. So, the main goal is to identify a set of genetic variants that happened together. Besides, it can determine individuals who must start screening at a young age (Chen et al., 2016). Determination of related genes to disease and disease mechanisms is a research goal and a necessary process. Some challenges are summarized in the following points, and also as shown in Fig. 10:

**Data processing:** The processing of large-scale DNA sequence data still presents efficiency challenges.

**Data Representation:** Certain problems exist with the quantification of DNA sequence aspects. No one knows which representation in these nucleotides is best for encoding numeric values. However, we cannot avoid using the numeric representations of those biological units when applying ML to biological studies.

**Genes and gene pairs:** A new method is required to discover associated genes and gene pairs or to study the relation between genes with subgroups of disorders. Recent discoveries about the new sub-classification of AD patients make this disease a suitable target for this method. To verify their significance, autism candidate genes identified in this study should be integrated into future data collection. These genes are contributed by understanding the mechanisms of genes to develop autism subtypes (Jain, Jain & Jain, 2018).

**Identification of genetic risk variants:** Identifying genetic risk variants in ASD and their effects on the brain and morphology remains largely elusive. More recently, the advent of a gene imaging approach integrates genetic discoveries with technological advances in brain imaging. Researchers were strongly encouraged to analyze ASD pathophysiology mechanisms and to characterize neurological systems which are directly affected by risk gene variants (Jain, Jain & Jain, 2018; Spencer et al., 2018).

**Heterogeneity in expression levels across large samples:** Due to heterogeneity, there may be an asymmetry in this effect on sample quality among patients with genetic brain disease and the degree of affected gene expression. Recently, an attempt was made to identify heterozygous genes in the study of gene expression data with genetic brain disease patients (Tejeswinee, Shomona & Athilakshmi, 2017, Escott-Price et al., 2014; Baker et al., 2019; Ruiz et al., 2014; Edwards, Stajich & Hansen, 2009; Staples et al., 2019; Pop & Salzberg, 2008; Yazdani et al., 2018).

**Linking genetics and brain imaging:** The genetic imaging techniques make it possible to improve our understanding of the etiology of genetic diseases by bridging the gap between genetic differences and their resulting biological effects on the brain (Chu & Wang, 2005; Hussain et al., 2019).

**The capability of generalizing**: The ability to generalize and adapt techniques to various data is considered the most challenging task. Also, the ability to reduce hardware power requirements is considered a critical task.

**Genetic structure for rare diseases and common complex diseases:** Rare and common diseases may be used for the study of Next Generation Sequencing (NGS). Causal variants can be found even in smaller sample sizes for single-gene rare diseases. It doesn’t remain easy, however, above all to define causal alleles. Not all rare diseases have a simple genetic structure like single-gene diseases. Rare diseases are usually diagnosed or identified by symptoms, while different mechanisms can cause the same symptoms. In fact, some rare diseases are a group of diseases that exhibit similar symptoms (Gao & Tembine, 2016). Determination of causal variants of these diseases generally requires larger sample sizes than monogenic diseases. NGS technologies provide a huge quantity of biological data, presenting different problems, including high processing times and high memory requirements. Research is therefore aimed at detecting SNP in genome sequences which can solve these problems. Also, there are many problems with SNPs detection algorithms, *e.g*., overhead computing costs, accuracy, and memory requirements. These issues are considered as another challenge for SNP identification (Tahir & Sardaraz, 2020; Wang, Lu & Zhao, 2015).

In the future, researchers should focus on identifying variations of rare diseases. They have great potential in the last few years, and genotyping and sequencing technologies have advanced. The researchers should also concentrate on these discoveries, the technologies that supported their discovery of rare variant detection and working with big data as challenges. Another challenge is imaging genetics, which addresses this question by bridging the gap between imaging and the genetic fields. Finally, finding the relationship between genes also helps discover complex diseases that are affected by risk genes.

In the future, we need to learn how to use biomarkers together in an integrated manner and learn to map them to specific necessary treatment actions or symptoms. Biomarkers may also be very helpful in explaining the significant variability associated with genetic diseases. The underlying pathophysiology symptoms of genetic diseases may vary significantly from patient to patient. ML is the DM core and the most widely used method of data processing. The main advantage of ML algorithms is that it can be used to filter large quantities of data to check patterns which could be ignored otherwise. ML plays a crucial role in discovering predictable patterns in biological systems and big data biomedical research. The current implementation of ML in biomedical data mainly faces the following issues:

1. Big datasets are essential to ML. Currently, most biological datasets are still too small to meet the requirements of ML algorithms. Although the total amount of biological data is huge and increases daily, data collection is obtained from various platforms. Due to technology and biology differences themselves, it is challenging for different data sets to integrate.
2. Because of biological data differences itself, trained ML models on one dataset may not generalize well to other datasets. The results of the analysis of the ML model can be wrong if the new data is significantly different from the training data.
3. The black-box nature of ML models obtained a new challenge to biological applications. The translation of the output of a specific model from a biological viewpoint that limits the use of the model is usually difficult.
4. Reduction of computational requirements: ML models are often very complex and require a lot of training. It is often computer-intensive and memory-intensive to produce well-trained modeling and even to use the model productively. These requirements limit the deployment of ML seriously on computer-saving machines, especially in the bioinformatics and healthcare sectors, which are also data-intensive. Several methods were proposed for ML compression, which can at the very beginning reduce the computational requirements of these models, like parameter pruning, which reduces the redundant parameters that do not contribute to the performance of the model.
5. Achieving the best performance and perfect classification by implementing various optimization techniques.
6. Additional attempts could be made to extract other DNA features before feeding them to the models. Overall, this could lead to more accurate assessments.

Currently, many softwares can be helpful for ML, and knowledge discovery tools for different uses are available, such as the Waikato Environment for Knowledge Analysis (WEKA), RapidMiner, Clementine, Intelligent Miner, Rosetta, etc. These tools and software provide methods and algorithms that help users better use information and data, including data analysis methods and algorithms, cluster analysis, etc (Borges, Marques & Bernardino, 2013).

**RapidMiner (RM)**: It is an ML and DM processing environment. It is an open-source, free Java project. It is a new way of designing all the complicated problems. For the input and output of data in various file formats, RM has flexible operators. It includes over 100 learning schemes for regression, classification, and clustering tasks (Borges, Marques & Bernardino, 2013).

**WEKA**: It is an open-source, non-commercial project. WEKA includes tools for data preprocessing, classification, regression, clustering, association rules, and visualization. It is also ideal for developing new systems of ML (Borges, Marques & Bernardino, 2013).

**R**: It is one of the key data science languages. It offers excellent visualization features that are essential to explore the data in an automated learning process before it is submitted and the results of the learning algorithm assessed. A lot of R packages are available for ML, and R implements numerous modern methods for statistical learning (Borges, Marques & Bernardino, 2013).

So, ML models will in the future offer many opportunities for discovering new insights from the research gaps discussed. In particular, analysis of genetic variations that lead to diseases, such as ASD, AD, cancer, and other fatal diseases will help find out a way to cure new medicines and therapies permanently. As shown in Fig. 9, we present limitations and future directions to handle it in the future.

## Conclusion

This review comprehensively reviews genetic variations analysis of gene expression analysis to discover complex genes associated with diseases and related genetic diseases. This survey describes various diseases, such as AD, ASD, and cancer, to identify genetic variations that cause diseases. Here, we describe variation identified for putative rare genetic risk. We observe that variants’ rare identification can decrease disease susceptibility than generally seen with common risk variation and protein-coding changes that can be modeled efficiently. Finally, we have highlighted some open issues and future research directions for the progress of this field. Also, genetic mapping supplies a powerful method for identifying the presence of SNPs in genes. These SNPs are found in DNA, which is very similar to mutation. But this SNP is damaged DNA in humans, causing serious disease in the future. So, our focus is to detect the SNPs that lead to diseases. New genome-wide multiple gene testing or high-throughput genetic testing has come with new hope in the field, offering a fast, cost-effective, and intensive analysis of genetic variation. This is particularly interesting with high genetic disorders heterogeneity. Therefore, the interaction between data mining, machine learning, and bioinformatics should be increased as a great potential for analyzing gene sequences in the future.

## Additional Information and Declarations

### Data Availability

The following information was supplied regarding data availability: This literature review has no data.
